# Comprehensive Kinetic Survey of Intestinal, Extra-Intestinal and Systemic Sequelae of Murine Ileitis Following Peroral Low-Dose *Toxoplasma gondii* Infection

**DOI:** 10.3389/fcimb.2019.00098

**Published:** 2019-04-12

**Authors:** Markus M. Heimesaat, Ildiko R. Dunay, Stefan Bereswill

**Affiliations:** ^1^Institute of Microbiology, Infectious Diseases and Immunology, Charité - University Medicine Berlin, Corporate Member of Freie Universität Berlin, Humboldt-Universität zu Berlin, and Berlin Institute of Health, Berlin, Germany; ^2^Medical Faculty, Institute of Inflammation and Neurodegeneration, University Hospital Magdeburg, Magdeburg, Germany

**Keywords:** subacute and chronic ileitis, low-dose *Toxoplasma gondii* infection, Th1-type immunopathology, gut microbiota changes, host-pathogen-interactions

## Abstract

We have recently shown that following peroral low-dose *Toxoplasma gondii* infection susceptible mice develop subacute ileitis within 10 days. Data regarding long-term intestinal and extra-intestinal sequelae of infection are scarce, however. We therefore challenged conventional C57BL/6 mice with one cyst of *T. gondii* ME49 strain by gavage and performed a comprehensive immunopathological survey 10, 36, and 57 days later. As early as 10 days post-infection, mice were suffering from subacute ileitis as indicated by mild-to-moderate histopathological changes of the ileal mucosa. Furthermore, numbers of apoptotic and proliferating/regenerating epithelial cells as well as of T and B lymphocytes in the mucosa and lamina propria of the ileum were highest at day 10 post-infection, but declined thereafter, and were accompanied by enhanced pro-inflammatory mediator secretion in ileum, colon and mesenteric lymph nodes that was most pronounced during the early phase of infection. In addition, subacute ileitis was accompanied by distinct shifts in the commensal gut microbiota composition in the small intestines. Remarkably, immunopathological sequelae of *T. gondii* infection were not restricted to the intestines, but could also be observed in extra-intestinal tissues including the liver, kidneys, lungs, heart and strikingly, in systemic compartments that were most prominent at day 10 post-infection. We conclude that the here provided long-term kinetic survey of immunopathological sequalae following peroral low-dose *T. gondii* infection provides valuable corner stones for a better understanding of the complex interactions within the triangle relationship of (parasitic) pathogens, the host immunity and the commensal gut microbiota during intestinal inflammation. The low-dose *T. gondii* infection model may be applied as valuable gut inflammation model in future pre-clinical studies in order to test potential treatment options for intestinal inflammatory conditions in humans.

## Introduction

Within 1 week following peroral high-dose infection with more than 50 cysts of the intracellular protozoan parasite *Toxoplasma gondii* strain ME49 susceptible mice irrespective whether harboring a murine or human gut microbiota develop acute necrotizing inflammation of the terminal ileum with lethal outcome (Heimesaat et al., 2006; Munoz et al., 2011; von Klitzing et al., 2017a,b). This CD4+ T lymphocyte dependent inflammatory condition is characterized by massive secretion of pro-inflammatory mediators such as TNF, IFN-γ, and nitric oxide, whereas IL-10 expression is upregulated as counter-regulatory measure (Liesenfeld et al., 1996, 1999; Jankovic et al., 2010; Munoz et al., 2011). Given the terminal ileum as predilection site, the underlying Th1-type immunopathology, and the pronounced changes in the commensal gut microbiota composition (i.e., dysbiosis) characterized by an overgrowth of the inflamed ileal lumen with commensal *Escherichia coli* and *Bacteroides / Prevotella* species, the high-dose *T. gondii* infection model mimics key features of human inflammatory bowel diseases such as Crohn's disease (Liesenfeld, 2002; Heimesaat et al., 2006; Munoz et al., 2009, 2011, 2015). However, data regarding long-term intestinal and extra-intestinal sequelae of peroral murine *T. gondii* post-infection resulting in intestinal inflammation are lacking due to the fatal immunopathological course within 1 week post infection (p.i).

We have previously established a subacute and non-lethal ileitis model following peroral low-dose (i.e., one cyst) *T. gondii* ME49 strain infection (Dunay et al., 2008; Escher et al., 2018; Heimesaat et al., 2018a). In order to further dissect the interplay between parasite, host immunity and gut microbiota (the latter mimicking human conditions) during infection-induced inflammation we had generated mice harboring a complex human gut microbiota prior ileitis induction (Bereswill et al., 2011; von Klitzing et al., 2017a,b,c,d; Escher et al., 2018; Heimesaat et al., 2018a,b). Within 9 days p.i. mice were suffering from T cell dependent subacute ileitis that was characterized by rather mild symptoms and mild-to-moderate ileal histopathological changes without necrosis (Escher et al., 2018; Heimesaat et al., 2018a). We did, however, not follow-up mice suffering from subacute ileitis beyond day 9 p.i.

This prompted us in the present study to perform a comprehensive long-term immunopathological survey of conventional C57BL/6j mice until day 57 following ileitis induction. We here demonstrate that immunopathological sequelae of ileitis induction (i) were most severe during the early phase of peroral low-dose *T. gondii* infection (i.e., around day 10 p.i.), (ii) affected not only the terminal ileum but also the colon, (iii) were accompanied by distinct shifts in the commensal microbiota composition within the ileal lumen, and (iv) were not restricted to the intestinal tract but could also be observed in extra-intestinal and (v) strikingly, even in systemic compartments.

## Materials and Methods

### Ethics Statement

Mouse experiments were performed in accordance with the European Guidelines for animal welfare (2010/63/EU). The protocols for these experiments were approved by “Landesamt für Gesundheit und Soziales,” LaGeSo, Berlin (registration numbers G244/98). Clinical conditions of mice were assessed at least once daily.

### Mice and *T. gondii* Infection

C57BL/6j mice were reared and housed under specific pathogen free (SPF) conditions in the Forschungseinrichtungen für Experimentelle Medicine (Charité—University Medicine Berlin). Conventionally colonized female mice 12 weeks of age were included into the experiments. On day (d) 0, mice were perorally subjected to low-dose *T. gondii* infection (i.e., one cyst of strain ME49 in a volume of 0.3 mL by gavage) as described recently (Heimesaat et al., 2006, 2018a). *T. gondii* DNA was quantitated in homogenized ileal tissue (~1 cm^2^) as stated elsewhere (Munoz et al., 2009).

### Sampling Procedures

At defined time points, namely d10, d36, or d57 p.i., mice were sacrificed by isofluran inhalation (Abbott, Germany). Naive mice served as uninfected controls. Upon necropsy, cardiac blood, luminal ileal samples as well as *ex vivo* biopsies from mesenteric lymph nodes (MLN), spleen, liver, kidneys, lungs, heart, colon and ileum were obtained under sterile conditions.

### Histopathology

After removal ileal *ex vivo* biopsies were immediately fixed in 5% formalin and embedded in paraffin. After hematoxylin and eosin (H&E) staining, histopathological changes were quantitatively assessed in 5 μm thin sections applying a standardized scoring system ranging from 0 to 6 as stated elsewhere (Heimesaat et al., 2006).

## Immunohistochemistry

For *in situ* immunohistochemical analyses *ex vivo* biopsies were obtained from the ileum, colon, liver, kidneys, lungs and the heart, immediately fixed in 5% formalin and embedded in paraffin. In order to quantitatively determine apoptotic and proliferating epithelial cells, macrophages and monocytes, T lymphocytes and B lymphocytes cells, paraffin sections (5 μm) were stained with primary antibodies directed against cleaved caspase 3 (Asp175, Cell Signaling, Beverly, MA, USA, 1:200), Ki67 (TEC3, Dako, Denmark, 1:100), F4/80 (# 14-4801, clone BM8, eBioscience, San Diego, CA, USA, 1:50), CD3 (#N1580, Dako, 1:10), and B220 (No. 14-0452-81, eBioscience; 1:200), respectively, as described earlier (Heimesaat et al., 2018b). Positively stained cells were then determined applying light microscopy (magnification 100 x and 400 x), and for each mouse the average number of respective positively stained cells was assessed within at least six high power fields (HPF, 0.287 mm^2^, 400 x magnification) by a blinded independent investigator. In order to provide a broader overview of the *T. gondii* induced immunopathological sequelae, representative photomicrographs of the immunohistochemically stained paraffin sections are provided with lower magnification (100 x) in the Online Supplemental Materials.

### Pro- and Anti-inflammatory Mediator Detection

Intestinal *ex vivo* biopsies were cut longitudinally, washed in phosphate buffered saline (PBS; Gibco, Life Technologies, UK), and strips of ~1 cm^2^ tissue as well as *ex vivo* biopsies derived from MLN (3 lymph nodes), liver (~1 cm^3^), one kidney (cut longitudinally), one lung, and spleen (one third) were transferred to 24-flat-bottom well-culture plates (Nunc, Germany) containing 500 μL serum-free RPMI 1640 medium (Gibco, life technologies, UK) supplemented with penicillin (100 U/mL) and streptomycin (100 μg/mL; PAA Laboratories, Germany). After 18 h at 37°C, respective culture supernatants as well as serum samples were tested for TNF, IFN-γ, MCP-1, IL-6, IL-12p70, and IL-10 by the Mouse Inflammation Cytometric Bead Assay (CBA; BD Biosciences, Germany) on a BD FACSCanto II flow cytometer (BD Biosciences). Nitric oxide was measured by the Griess reaction as stated elsewhere (Heimesaat et al., 2006).

### Gut Microbiota Analyses

DNA was extracted from ileal luminal samples as reported recently (Heimesaat et al., 2006; Bereswill et al., 2014). In brief, DNA was quantitated applying the Quant-iT PicoGreen reagent (Invitrogen, UK) and adjusted to 1 ng per μL. The total eubacterial load as well as the main bacterial groups within the murine gut microbiota such as enterobacteria, enterococci, lactobacilli, bifidobacteria, *Bacteroides / Prevotella* species, *Clostridium coccoides* group, and *Clostridium leptum* group were determined by quantitative real-time polymerase chain reaction (qRT-PCR) with species-, genera- or group-specific 16S rRNA gene primers (Tib MolBiol, Germany) as stated elsewhere (Heimesaat et al., 2010; Bereswill et al., 2011; Rausch et al., 2013) (expressed as numbers of 16S rRNA gene copies per ng DNA).

### Statistical Analysis

Medians and levels of significance were determined by one-way ANOVA or Kruskal-Wallis test followed by Tukey post-correction for multiple comparisons (GraphPad Prism v7, USA) as indicated. Two-sided probability (p) values ≤ 0.05 were considered significant. Experiments were reproduced three times.

## Results

### Intestinal Inflammatory Changes Upon Peroral Low-Dose *T. gondii* Infection

**Figure 1:**
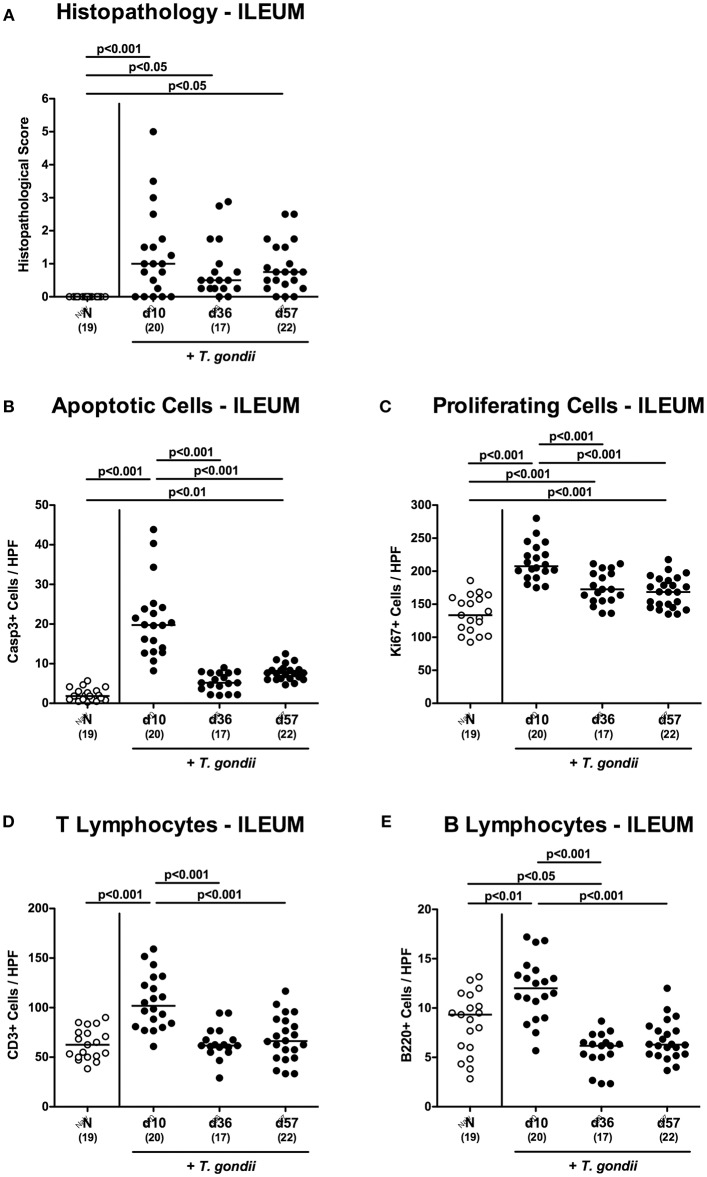
Microscopic inflammatory sequelae in the ileum over time following peroral low-dose *T. gondii* infection. Mice were perorally infected with one cyst of *T. gondii* on day 0 and surveyed at days (d) 10, 36, and 57 post-infection (p.i.; closed circles). Naive (N) mice served as uninfected controls (open circles). **(A)** Histopathological changes were quantified in H&E stained paraffin sections from ileal *ex vivo* biopsies assessing a standardized histopathological scoring system. Out of six representative high power fields (HPF, 400x magnification) per animal the average numbers of **(B)** apoptotic (casapse3+, Casp3+) and **(C)** proliferating (Ki67+) ileal epithelial cells as well as of **(D)** T lymphocytes (CD3+) and **(E)** B lymphocytes within the ileal mucosa and lamina propria were assessed in immunohistochemically stained small intestinal paraffin sections. Medians (black bars), levels of significance (*p*-values) as determined by one-way ANOVA test followed by Tukey post-correction for multiple comparisons and numbers of analyzed mice (in parentheses) are indicated. Data were pooled from four independent experiments.

In order to induce subacute ileitis, conventionally colonized mice were perorally subjected to low-dose (i.e., one cyst of) *T. gondii* challenge by gavage and to a comprehensive immunopathological survey for almost 2 months p.i. Within 10 days upon infection mild-to-moderate histopathological changes of the ileal mucosa could be observed ranging from edematous mucosal blubbing, leukocytic infiltrates of the ileal mucosa and lamina propria, cell-free exudates into the small intestinal lumen, and cellular shedding (*p* < 0.001 vs. naive; Figure 1A and Figure S1A). As compared to day 10 p.i., median histopathological scores quantitatively assessing ileal mucosal damage tended to be lower at later time points, but did not reach statistical significance due to high standard deviations (n.s.; Figure 1A and Figure S1A). We additionally quantitatively surveyed intestinal inflammatory responses applying *in situ* immunohistochemistry of intestinal paraffin sections. In *T. gondii* infected mice, multifold increased apoptotic ileal epithelial cell numbers could be determined at days 10 and 57 (*p* < 0.001 and *p* < 0.01, respectively vs. naive) with highest counts in *ex vivo* biopsies taken at day 10 p.i. (*p* < 0.001 vs. day 36 and day 57 p.i.; Figure 1B and Figure S1B). In parallel, Ki67+ ileal epithelial cells indicative for cell proliferation and regeneration counteracting *T. gondii* induced cell damage increased to a maximum at day 10 p.i. (*p* < 0.001), declined thereafter, but were still higher as compared to those in naive control mice (*p* < 0.001; Figure 1C and Figure S1C).

Given that *T. gondii* induced ileitis is highly T cell dependent (Munoz et al., 2011), we quantitated CD3+ T lymphocytes in the ileal mucosa and lamina propria. In fact, T cell numbers almost doubled with 10 days p.i., but declined back to naive counts thereafter (*p* < 0.001; Figure 1D and Figure S1D). In addition, *T. gondii* infected mice also displayed elevated B cell numbers in their ileal mucosa and lamina propria at day 10 p.i. (*p* < 0.01; Figure 1E and Figure S1E), but not at later time points.

**Figure 2:**
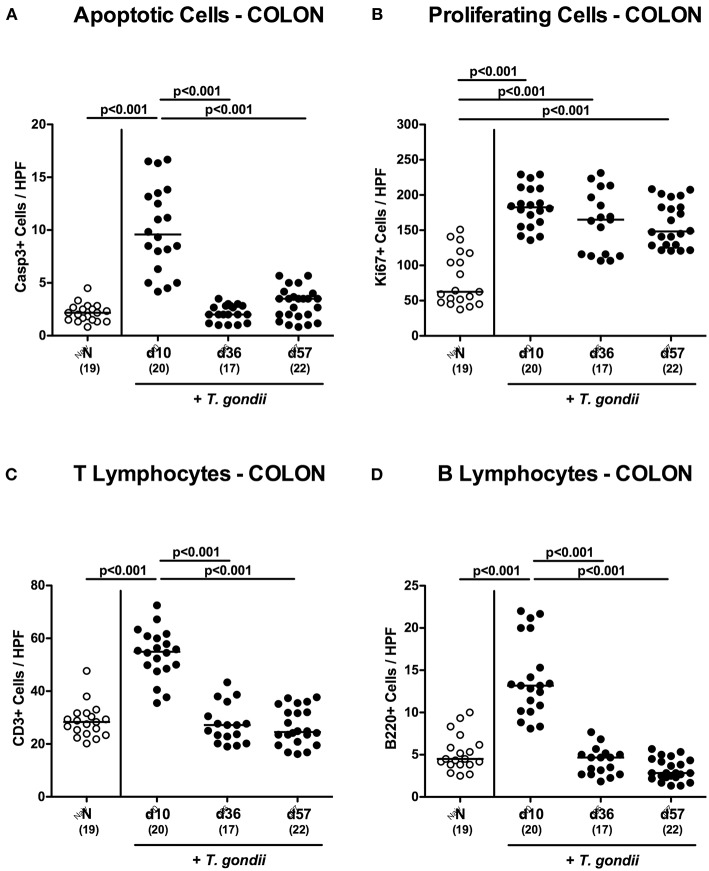
Microscopic inflammatory sequelae in the colon over time following peroral low-dose *T. gondii* infection. Mice were perorally infected with one cyst of *T. gondii* on day 0 and surveyed at days (d) 10, 36, and 57 post-infection (p.i.; closed circles). Naive (N) mice served as uninfected controls (open circles). Out of six representative high power fields (HPF, 400x magnification) per animal the average numbers of **(A)** apoptotic (casapse3+, Casp3+) and **(B)** proliferating (Ki67+) colonic epithelial cells as well as of **(C)** T lymphocytes (CD3+) and **(D)** B lymphocytes within the colonic mucosa and lamina propria were assessed in immunohistochemically stained large intestinal paraffin sections. Medians (black bars), levels of significance (*p*-values) as determined by one-way ANOVA test followed by Tukey post-correction for multiple comparisons and numbers of analyzed mice (in parentheses) are indicated. Data were pooled from four independent experiments.

We have recently observed that *T. gondii* induced immunopathological responses within the intestinal tract do not exclusively affect the terminal ileum, but also the large intestines (Escher et al., 2018; Heimesaat et al., 2018a) and therefore expanded our inflammatory survey to the colon. Histopathological mucosal changes within in colonic mucosa were rather minor, however (Figure S2A), and did not reach statistical significance due to high standard deviations within respective groups (n.s.; not shown). When quantitating apoptotic cells by *in situ* immunohistochemistry, multifold increased numbers of apoptotic colonic epithelial cells could be assessed only at day 10 p.i. (*p* < 0.001; Figure 2A and Figure S2B), which also held true for T lymphocytes (*p* < 0.001; Figure 2C and Figure S2D) and B lymphocytes in the large intestinal mucosa and lamina propria (*p* < 0.001; Figure 2D and Figure S2E). Like in the ileum, elevated Ki67+ cells numbers could be observed in the colonic epithelia upon *T. gondii* infection (*p* < 0.001; Figure 2B and Figure S2C),

Hence, apoptotic, proliferating and T cell as well as B cell responses within both, the small as well as the large intestines were most pronounced 10 days following peroral low-dose *T. gondii* infection.

### Intestinal Inflammatory Mediator Secretion Upon Peroral Low-Dose *T. gondii* Infection

**Figure 3:**
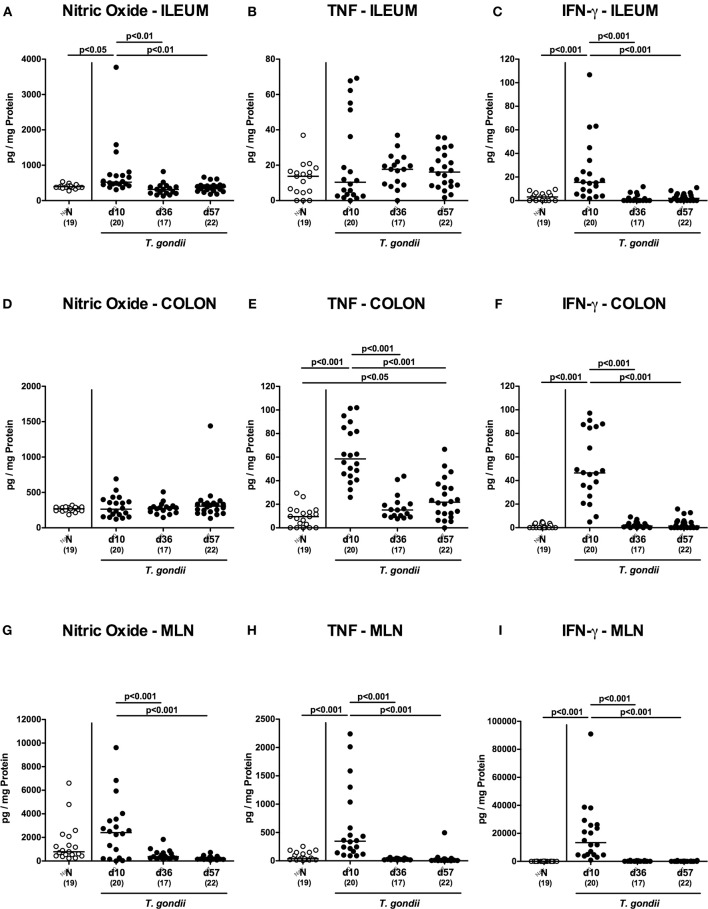
Intestinal pro-inflammatory mediator secretion over time following peroral low-dose *T. gondii* infection. Mice were perorally infected with one cyst of *T. gondii* on day 0 and surveyed at days (d) 10, 36, and 57 post-infection (p.i.; closed circles). Naive (N) mice served as uninfected controls (open circles). Pro-inflammatory mediators such as **(A,D,G)** nitric oxide, **(B,E,H)** TNF and **(C,F,I)** IFN-γ were measured in *ex vivo* biopsies taken from distinct intestinal compartments including the **(A–C)** ileum, **(D–F)** colon and **(G–I)** mesenteric lymph nodes (MLN) at respective time points. Medians (black bars), levels of significance (*p*-values) as determined by one-way ANOVA test followed by Tukey post-correction for multiple comparisons and numbers of analyzed mice (in parentheses) are indicated. Data were pooled from four independent experiments.

We next assessed pro-inflammatory mediator secretion in *ex vivo* biopsies derived from distinct intestinal compartments (Figure 3). At day 10 p.i., increased ileal nitric oxide and IFN-γ concentrations could be measured (*p* < 0.001; Figures 3A,C), that declined to naive levels thereafter. In the colon, however, increased TNF concentrations could be observed at either time point p.i. (*p* < 0.05-0.001; Figure 3E) with maximum levels at day 10 p.i. (*p* < 0.001; Figure 3E). Alike the ileum, increased IFN-γ concentrations were measured in large intestinal *ex vivo* biopsies exclusively at day 10 p.i. (*p* < 0.001; Figure 3F), whereas elevated colonic TNF levels could be measured at days 10 and 57 p.i. (*p* < 0.001 and *p* < 0.05, respectively; Figure 3E).

We further surveyed pro-inflammatory mediator secretion in MLN draining the inflamed intestines. As early as day 10 p.i., TNF and IFN-γ concentrations had increased in the MLN (*p* < 0.001; Figures 3H,I), but declined to naive levels later-on, which also held true for nitric oxide (*p* < 0.05–0.005; Figure 3G).

Hence, peroral low dose *T. gondii* infection was accompanied by pronounced pro-inflammatory mediator secretion in the entire intestinal tract, particularly during the early phase (i.e., the first 10 days p.i.).

### Intestinal Microbiota Changes Upon Peroral Low-Dose *T. gondii* Infection

**Figure 4:**
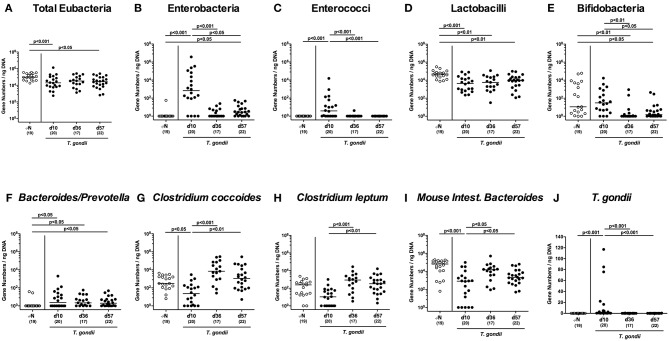
Ileal microbiota composition and parasitic loads over time following peroral low-dose *T. gondii* infection. Mice were perorally infected with one cyst of *T. gondii* on day 0 and surveyed at days (d) 10, 36, and 57 post-infection (p.i.; closed circles). Naive (N) mice served as uninfected controls (open circles). **(A)** The total eubacterial loads as well as the main gut bacterial groups (**B–I**, as indicated) and **(J)** *T. gondii* DNA were quantitated in ileal samples by culture-independent molecular methods and expressed as gene numbers per ng DNA. Medians (black bars), levels of significance (*p*-values) as determined by one-way ANOVA test followed by Tukey post-correction for multiple comparisons and numbers of analyzed mice (in parentheses) are indicated. Data were pooled from four independent experiments.

Given that inflammatory processes in the intestinal tract are associated with pronounced shifts in the gut microbiota composition (Heimesaat et al., 2006, 2007a,b; Erridge et al., 2010; Bereswill et al., 2011; Fiebiger et al., 2016), we quantitatively surveyed changes in distinct intestinal gut bacterial groups and species applying culture-independent 16S rRNA based methods (Figures 4A–I). Within 10 days p.i., enterobacteria and enterococci had increased in luminal samples taken from the inflamed ileum (*p* < 0.001; Figures 4B,C), but declined thereafter (*p* < 0.05–0.001; Figures 4B,C), whereas obligate anaerobic Gram-negative species such as *Bacteroides / Prevotella* species were slightly higher at either time point p.i. as compared to naive mice (*p* < 0.05; Figure 4F). Conversely, lactobacilli were lower in ileal samples derived from *T. gondii* infected mice (*p* < 0.01–0.001; Figure 4D), which also held true for bifidobacteria at days 36 and 57 p.i. (*p* < 0.05–0.01; Figure 4E). Converse to enterobacteria and enterococci, the obligate anaerobic *Clostridium coccoides* and *Mouse Intestinal Bacteroides* decreased to lowest levels until day 10 p.i. (*p* < 0.05–0.005; Figures 4G–I), but increased later-on (*p* < 0.05–0.001; Figures 4G,I). For *Clostridium leptum*, a trend toward lower gene numbers at day 10 p.i. as compared to naïve conditions could be observed (n.s. due to high standard deviations; Figure 4H), whereas bacterial loads were higher at days 36 and 37 vs. day 10 p.i. (*p* < 0.001 and *p* < 0.01, respectively; Figure 4H).

Of note, increased *T. gondii* DNA could be detected in small intestinal *ex vivo* biopsies at day 10 p.i., but not later-on (*p* < 0.001; Figure 4J).

Hence, *T. gondii* induced immunopathological sequelae observed in the ileum were accompanied by pronounced shifts in the luminal gut microbiota composition in the distal small intestines.

### Extra-Intestinal Inflammatory Changes Upon Peroral Low-Dose *T. gondii* Infection

**Figure 5:**
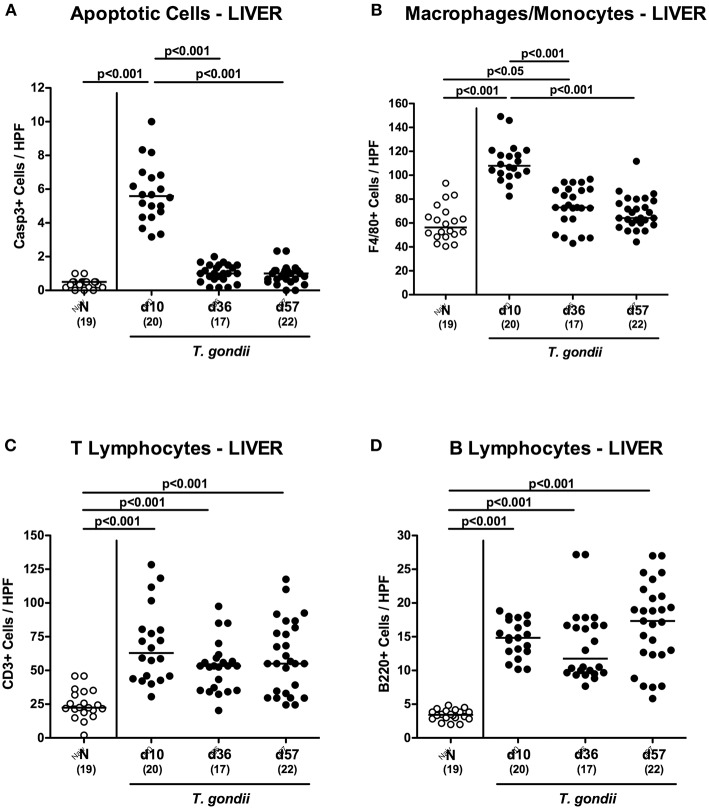
Microscopic inflammatory sequelae in the liver over time following peroral low-dose *T. gondii* infection. Mice were perorally infected with one cyst of *T. gondii* on day 0 and surveyed at days (d) 10, 36 and 57 post-infection (p.i.; closed circles). Naive (N) mice served as uninfected controls (open circles). Out of six representative high power fields (HPF, 400x magnification) per animal the average numbers of **(A)** apoptotic (casapse3+, Casp3+) and **(B)** proliferating (Ki67+) cells as well as of **(C)** T lymphocytes (CD3+) and **(D)** B lymphocytes were assessed in immunohistochemically stained hepatic paraffin sections. Medians (black bars), levels of significance (*p*-values) as determined by one-way ANOVA test followed by Tukey post-correction for multiple comparisons and numbers of analyzed mice (in parentheses) are indicated. Data were pooled from four independent experiments.

**Figure 6:**
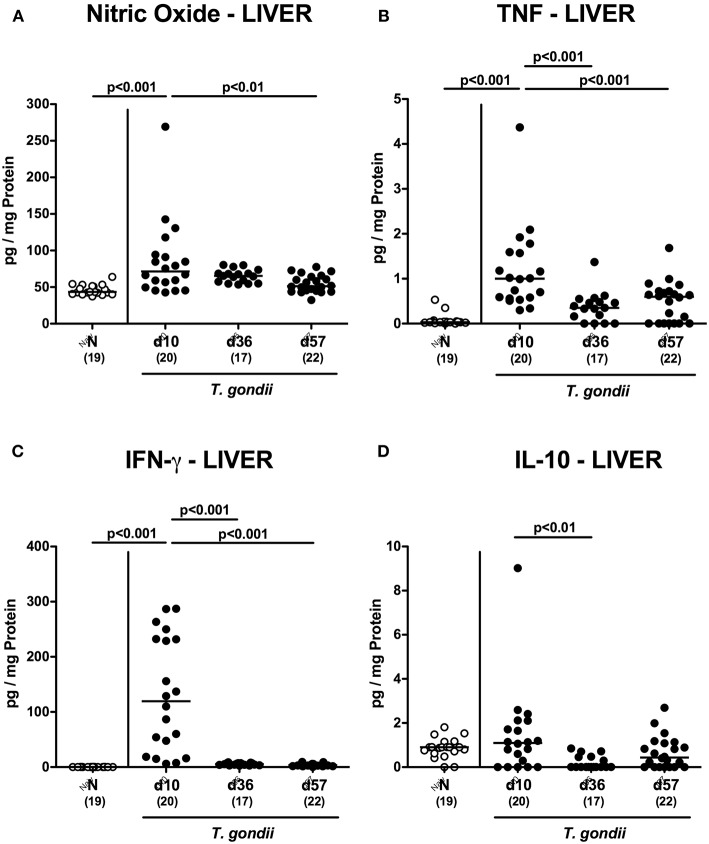
Hepatic inflammatory mediator secretion over time following peroral low-dose *T. gondii* infection. Mice were perorally infected with one cyst of *T. gondii* on day 0 and surveyed at days (d) 10, 36, and 57 post-infection (p.i.; closed circles). Naive (N) mice served as uninfected controls (open circles). **(A)** Nitric oxide, **(B)** TNF, **(C)** IFN-γ and **(D)** IL-10 concentrations were measured in *ex vivo* biopsies taken from the liver at respective time points. Medians (black bars), levels of significance (*p*-values) as determined by one-way ANOVA test followed by Tukey post-correction for multiple comparisons and numbers of analyzed mice (in parentheses) are indicated. Data were pooled from four independent experiments.

We next surveyed potential *T. gondii* induced inflammatory changes in extra-intestinal compartments over time. *T. gondii* infected mice displayed elevated numbers of caspase3+ cells in their livers exclusively at day 10 p.i. (*p* < 0.001; Figure 5A and Figure S4A). In addition, hepatic F4/80+ cells had increased as early as day 10 p.i. (*p* < 0.001), but declined to naive numbers until day 57 p.i. (*p* < 0.001; Figure 5B and Figure S3B). At either time point p.i., mice exhibited increased numbers of both, T and B lymphocytes in their livers (*p* < 0.001; Figures 5C,D and Figures S3C,D). Furthermore, *T. gondii* induced hepatic inflammatory cell responses were accompanied by enhanced secretion of pro-inflammatory mediators measured at day 10 p.i. (*p* < 0.001 vs. naive; Figures 6A–C). At day 36 p.i., hepatic anti-inflammatory IL-10 concentrations were lower as compared to those obtained at day 10 p.i. (*p* < 0.01; Figure 6D).

**Figure 7:**
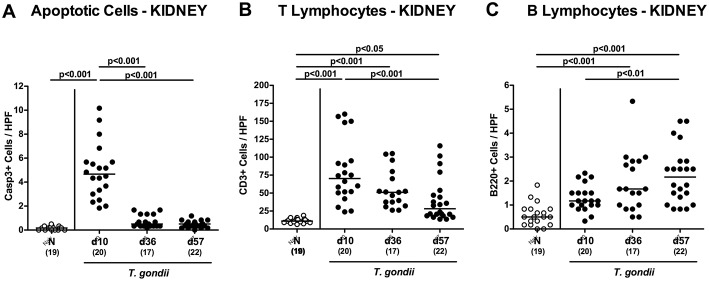
Microscopic inflammatory sequelae in the kidneys over time following peroral low-dose *T. gondii* infection. Mice were perorally infected with one cyst of *T. gondii* on day 0 and surveyed at days (d) 10, 36, and 57 post-infection (p.i.; closed circles). Naive (N) mice served as uninfected controls (open circles). Out of six representative high power fields (HPF, 400x magnification) per animal the average numbers of **(A)** apoptotic (casapse3+, Casp3+) cells, of **(B)** T lymphocytes (CD3+) and **(C)** B lymphocytes were assessed in immunohistochemically stained renal paraffin sections. Medians (black bars), levels of significance (*p*-values) as determined by one-way ANOVA test followed by Tukey post-correction for multiple comparisons and numbers of analyzed mice (in parentheses) are indicated. Data were pooled from four independent experiments.

**Figure 8:**
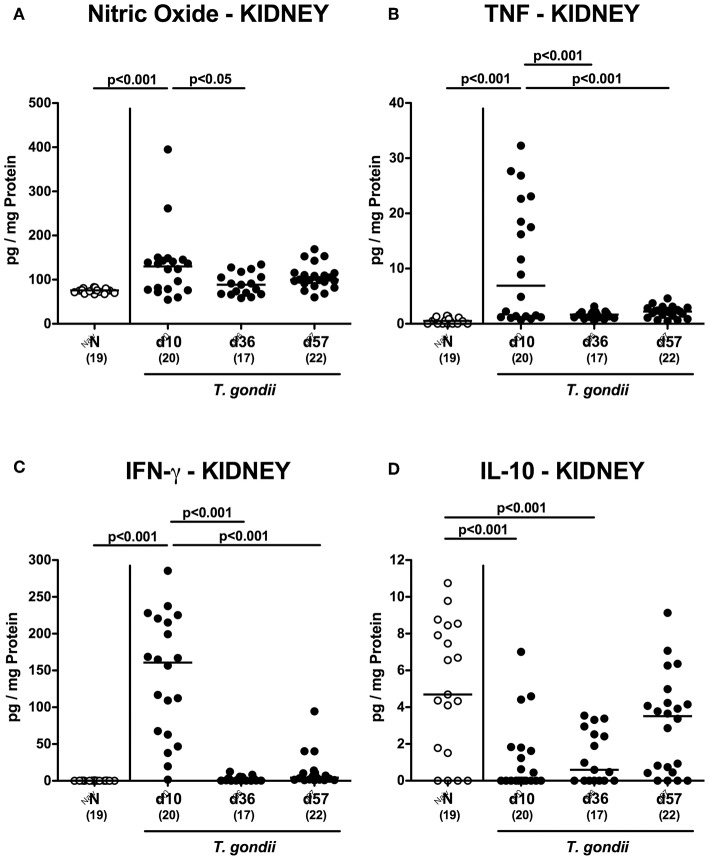
Renal inflammatory mediator secretion over time following peroral low-dose *T. gondii* infection. Mice were perorally infected with one cyst of *T. gondii* on day 0 and surveyed at days (d) 10, 36, and 57 post-infection (p.i.; closed circles). Naive (N) mice served as uninfected controls (open circles). **(A)** Nitric oxide, **(B)** TNF, **(C)** IFN-γ, and **(D)** IL-10 concentrations were measured in *ex vivo* biopsies taken from the kidneys at respective time points. Medians (black bars), levels of significance (*p*-values) as determined by one-way ANOVA test followed by Tukey post-correction for multiple comparisons and numbers of analyzed mice (in parentheses) are indicated. Data were pooled from four independent experiments.

Enhanced inflammatory responses upon low-dose *T. gondii* infection could also be observed in the kidneys. In fact, at day 10 following *T. gondii* infection mice exhibited multifold increased numbers of apoptotic cells in their kidneys, but not later-on (Figure 7A and Figure S4A), whereas renal T cell numbers were elevated at either time point p.i. (*p* < 0.05–0.001), but with lower counts at day 57 vs. day 10 p.i. (*p* < 0.001; Figure 7B and Figure S4B). Pathogen induced increases in renal B cell numbers could be observed rather late in the course of infection (i.e., days 36 and 57 p.i.; *p* < 0.001; Figure 7C and Figure S4C). When assessing pro-inflammatory mediator secretion in renal *ex vivo* biopsies, nitric oxide, TNF and IFN-γ increased as early as day 10 p.i. (*p* < 0.001; Figures 8A–C). Conversely, IL-10 concentrations were lower at days 10 and 36 p.i. when compared to naive mice (*p* < 0.001; Figure 8D).

**Figure 9:**
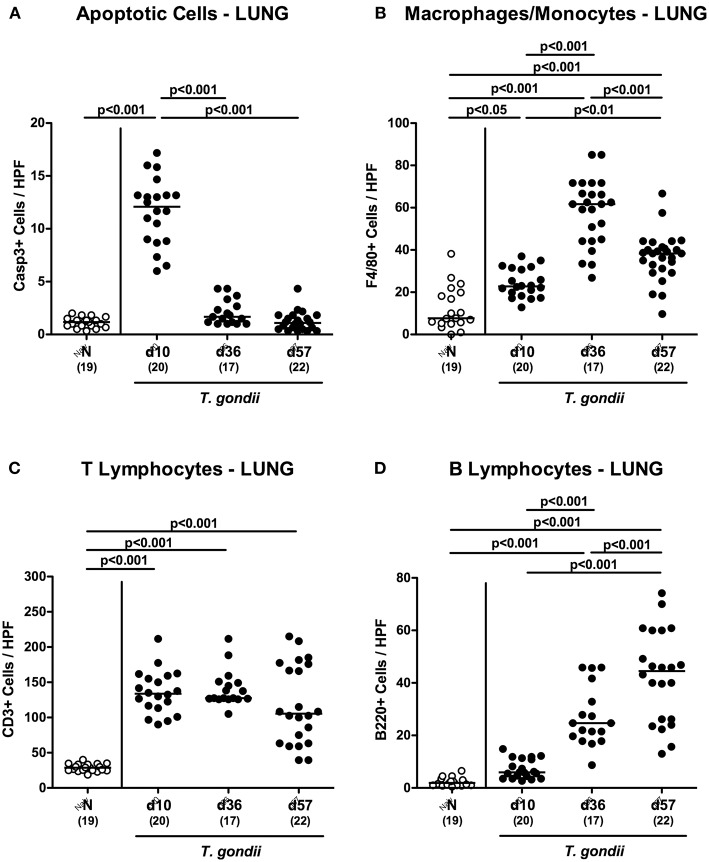
Microscopic inflammatory sequelae in the lungs over time following peroral low-dose *T. gondii* infection. Mice were perorally infected with one cyst of *T. gondii* on day 0 and surveyed at days (d) 10, 36, and 57 post-infection (p.i.; closed circles). Naive (N) mice served as uninfected controls (open circles). Out of six representative high power fields (HPF, 400x magnification) per animal the average numbers of **(A)** apoptotic (casapse3+, Casp3+) cells, of **(B)** macrophages and monocytes (F4/80+), of **(C)** T lymphocytes (CD3+) and **(D)** B lymphocytes were assessed in immunohistochemically stained pulmonary paraffin sections. Medians (black bars), levels of significance (*p*-values) as determined by one-way ANOVA test followed by Tukey post-correction for multiple comparisons and numbers of analyzed mice (in parentheses) are indicated. Data were pooled from four independent experiments.

We further expanded our inflammatory survey to the lungs. Apoptotic pulmonary cells dramatically increased until day 10 p.i. (*p* < 0.001), but decreased back to naïve levels later-on (*p* < 0.001; Figure 9A and Figure S5A). Low-dose *T. gondii* infected mice further exhibited increased numbers of F4/80+ cells in the lungs (*p* < 0.001) with maximum counts at day 36 p.i. (Figure 9B and Figure S5B). In addition, pulmonary T lymphocytes were elevated at either time point following *T. gondii* infection (*p* < 0.001; Figure 9C and Figure S5C), whereas *T. gondii* induced increases in B lymphocytes could be assessed in the later stages of the infection (i.e., days 36 and 57 p.i.; *p* < 0.001) with highest counts at the end of the survey; (*p* < 0.001; Figure 9D and Figure S5D).

**Figure 10:**
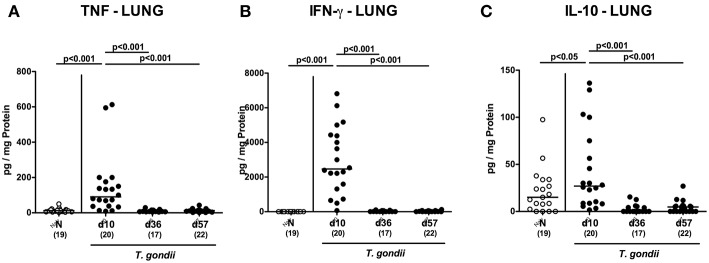
Pulmonal inflammatory mediator secretion over time following peroral low-dose *T. gondii* infection. Mice were perorally infected with one cyst of *T. gondii* on day 0 and surveyed at days (d) 10, 36, and 57 post-infection (p.i.; closed circles). Naive (N) mice served as uninfected controls (open circles). **(A)** TNF, **(B)** IFN-γ, and **(C)** IL-10 concentrations were measured in *ex vivo* biopsies taken from the lungs at respective time points. Medians (black bars), levels of significance (*p*-values) as determined by one-way ANOVA test followed by Tukey post-correction for multiple comparisons and numbers of analyzed mice (in parentheses) are indicated. Data were pooled from four independent experiments.

Interestingly, increased secretion of pro-inflammatory cytokines such as TNF and IFN-γ (*p* < 0.001) as well as of anti-inflammatory IL-10 concentrations (*p* < 0.05) could be exclusively determined in lungs taken at the earliest time point p.i. (Figure 10).

Remarkably, overt *T. gondii* induced inflammatory changes could also be assessed in the heart as indicated by increased numbers of apoptotic cells at days as well as of T lymphocytes in the cardiac muscle of infected mice (*p* < 0.01–0.001; Figures S6A,B, S7A,B), whereas highest apoptotic cell numbers could be counted at the end of the survey (*p* < 0.05 vs. d10 p.i.; Figures S6A, S7A). Furthermore, cardiac B cell numbers were higher on days 36 and 57 p.i. as compared to earlier time points (*p* < 0.05–0.01; Figures S6C, S7C).

Hence, pronounced inflammatory responses following peroral low-dose *T. gondii* infection were not restricted to the intestinal tract, but could also be observed in extra-intestinal compartments.

### Systemic Inflammatory Responses Upon Peroral Low-Dose *T. gondii* Infection

**Figure 11:**
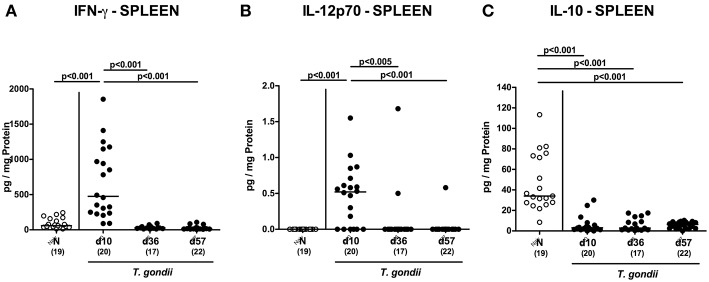
Splenic inflammatory mediator secretion over time following peroral low-dose *T. gondii* infection. Mice were perorally infected with one cyst of *T. gondii* on day 0 and surveyed at days (d) 10, 36, and 57 post-infection (p.i.; closed circles). Naive (N) mice served as uninfected controls (open circles). **(A)** IFN-γ, **(B)** IL-12p70, and **(C)** IL-10 concentrations were measured in *ex vivo* biopsies taken from the spleen at respective time points. Medians (black bars), levels of significance (*p*-values) as determined by one-way ANOVA test followed by Tukey post-correction for multiple comparisons and numbers of analyzed mice (in parentheses) are indicated. Data were pooled from four independent experiments.

**Figure 12:**
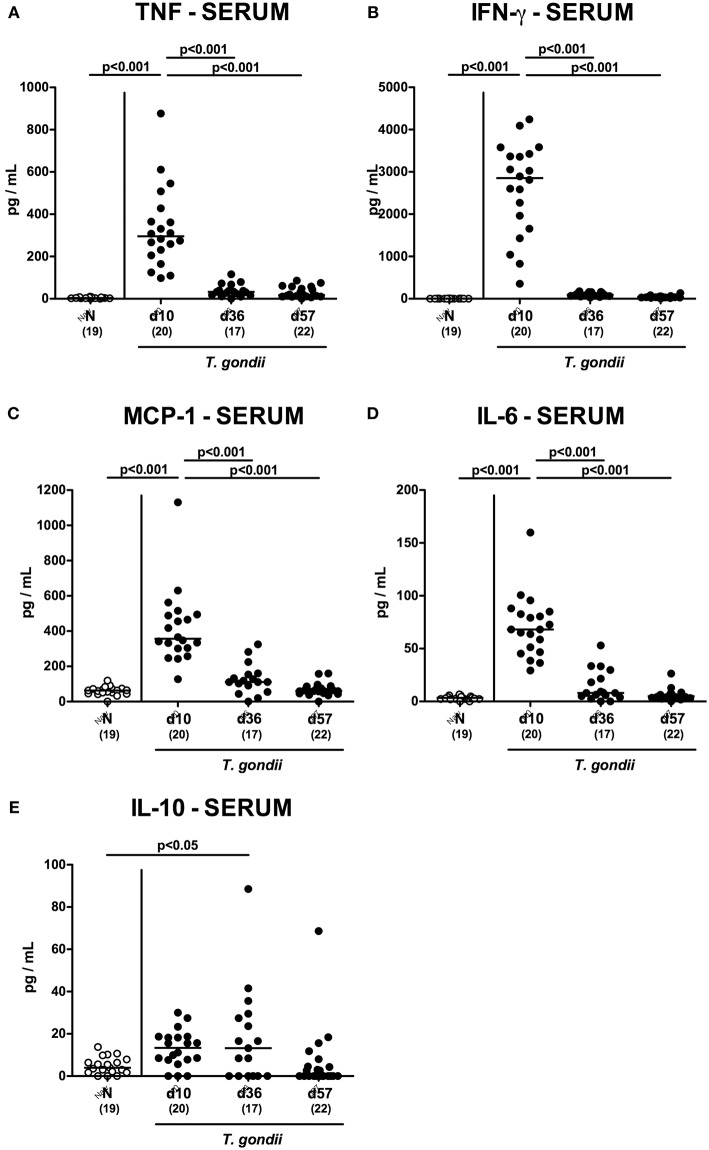
Systemic inflammatory mediator secretion over time following peroral low-dose *T. gondii* infection. Mice were perorally infected with one cyst of *T. gondii* on day 0 and surveyed at days (d) 10, 36, and 57 post-infection (p.i.; closed circles). Naive (N) mice served as uninfected controls (open circles). **(A)** TNF, **(B)** IFN-γ, **(C)** MCP-1, **(D)** IL-6 and **(E)** IL-10 concentrations were measured in serum samples taken at respective time points. Medians (black bars), levels of significance (*p*-values) as determined by one-way ANOVA test followed by Tukey post-correction for multiple comparisons and numbers of analyzed mice (in parentheses) are indicated. Data were pooled from four independent experiments.

We next assessed *T. gondii* induced immunopathological responses in systemic compartments. Whereas, increased pro-inflammatory cytokines such as IFN-γ and IL-12p70 could be measured in splenic *ex vivo* biopsies at day 10 p.i. only (*p* < 0.001; Figures 11A,B), anti-inflammatory IL-10 levels were deprived in the spleen at either time point p.i. (*p* < 0.001; Figure 11C). Strikingly, elevated TNF, IFN-γ, MCP-1 and IL-6 concentrations were obtained in serum samples taken from *T. gondii* infected mice at day 10 p.i. (*p* < 0.001), but declined back to baseline levels later-on (*p* < 0.001; Figures 12A–D). In case of IL-10, slightly increased serum concentrations could be exclusively observed at day 36 p.i. (*p* < 0.05; Figure 12E).

Hence, inflammatory responses following peroral low-dose *T. gondii* infection could be assessed even in systemic compartments that were most pronounced within the first 10 days p.i.

## Discussion

The peroral high-dose *T. gondii* infection mouse model has been proven to be well-suited for investigating the underlying molecular mechanism of pathogen-host interactions and subsequently induced acute small intestinal inflammation (McLeod et al., 1989a,b; Liesenfeld et al., 1996, 1999; Liesenfeld, 2002; Vossenkamper et al., 2004; Heimesaat et al., 2006; Munoz et al., 2009, 2011, 2015). In this context one needs to take into consideration, however, that the severity of *T. gondii* induced ileitis does not meet a linear causal relationship to the amount of parasite cysts the mice had been challenged with. In fact, it is rather a critical threshold of cyst number that needs to be reached to induce the full-blown inflammatory scenario, further depending on the parasitic strain and the genetic background and sex of the murine host (Liesenfeld et al., 1999; Munoz et al., 2011; Heimesaat et al., 2018a). Given the lethal outcome within 7–10 days, information regarding long-term sequelae of *T. gondii* ME49 strain induced intestinal inflammation are scarce, however.

We have established a far less acute *T. gondii* induced ileitis model by decreasing the numbers of the perorally applied parasitic infection dose from >50 cysts to only one cyst per recipient mouse (Dunay et al., 2008; Heimesaat et al., 2018a). One can imagine that an accidental increase in the parasitic inoculum may yield an unwantedly more severe ileitis with fatal outcome within <2 weeks. Besides the critically low infection dose, distinct host characteristics such as age and sex of the infected mice might impact the severity of induced immunopathology (Liesenfeld et al., 1999; Dunay et al., 2008; Munoz et al., 2011; Heimesaat et al., 2018a). Hence, the reproducibility and reliability of the peoral low-dose *T. gondii* infection model critically depends on highly standardized conditions and the experiences of the handling researchers (Heimesaat et al., 2018a). However, with the subacute ileitis model at hand, we are now able to survey the interactions within the triangle relationship (“ménage-à-trois”) of the parasitic pathogen, the host immunity and the commensal gut microbiota during intestinal inflammation for 2 months (like in the present study) or even longer. In our very recent low-dose *T. gondii* infection studies mice were sacrificed at day 9 p.i. (Escher et al., 2018; Heimesaat et al., 2018a).

In our actual immunopathological survey for more than 8 weeks, perorally low-dose *T. gondii* infected mice developed subacute ileitis within 10 days that was characterized by a non-lethal outcome, rather mild symptoms and mild-to-moderate histopathological changes of the ileal mucosa, but no intestinal necrosis at all. Furthermore, ileal epithelial apoptosis was accompanied by a concomitant increased abundance of proliferating/regenerating epithelial cells in the terminal ileum counteracting the inflammatory damage. Remarkably, *T. gondii* induced inflammatory changes did not exclusively affect the terminal ileum which is considered the predilection site following peroral high-dose *T. gondii* infection (Liesenfeld et al., 1996; Munoz et al., 2011), but also the large intestinal tract given that numbers of apoptotic epithelial cells as well as of T and B lymphocytes in the mucosa and lamina propria of both, the ileum and the colon were highest at day 10 post-infection, but declined thereafter. These inflammatory cellular responses were accompanied by enhanced pro-inflammatory mediator secretion in ileum, colon and mesenteric lymph nodes that was also most pronounced during the early phase of infection. In support, in our previous acute ileitis studies in (with respect to their gut microbiota) “humanized” mice we could demonstrate the colonic co-affection upon peroral high-dose *T. gondii* infection (von Klitzing et al., 2017a,b). Furthermore, Suzuki and colleagues reported inflammatory changes within the large intestines following peroral infection of IL-10^−/−^ mice with 20 cysts of the ME49 strain (Suzuki et al., 2000).

Remarkably, both peroral high-dose and low-dose *T. gondii* infection resulted in pro-inflammatory sequelae that were not restricted to the intestinal tract, but could also be observed in extra-intestinal and even systemic compartments as shown in our previous reports (von Klitzing et al., 2017a,b; Heimesaat et al., 2018a,b) and actual study. Overall, *T. gondii* induced and T cell driven pro-inflammatory immune responses were observed in extra-intestinal organs such as liver, kidneys, lungs, and heart during the entire observation period, but were overall most pronounced during the early phase (i.e., around day 10 p.i.). Depending on respective parasitic infection (i.e., amount and strain of cysts, application via the peroral vs. intraperitoneal route) of mice with a defined genetic background, sampling and detection techniques varying information regarding extra-intestinal parasitic infection are available in the literature. For instance, in one study exclusively assessing the parasitic dissemination following peroral infection with 20 cysts of the avirulent *T. gondii* C strain, parasitic loads had rapidly increased in the lungs until days 7 to 10 and resolved until day 50 p.i. (Derouin and Garin, 1991). Compared to pulmonary parasitic detection, parasitemia was rather delayed during the acute phase of infection (i.e., first 7 days p.i.), whereas low-level parasitemia was shown to occur transiently later-on (Derouin and Garin, 1991). In another infection study, *T. gondii* could be detected in liver and lungs of mice as early as 7-10 days following peroral challenge with 20 cysts of the avirulent C strain (Sumyuen et al., 1995). Dubey reported parasitemia within 24 h post peroral infection and parasitic invasion of extra-intestinal organs including liver, lungs, kidneys, skeletal and heart muscle between days 11 and 15 p.i. (Dubey, 1997). In these studies, however, no further information regarding immunopathological sequelae within infected compartments are available. Following challenge of C57BL/6 mice with 10 cysts of the ME49 strain via the intraperitoneal route, Liesenfeld and colleagues reported rather mild infiltration of lungs with inflammatory mononuclear cells within 7 days p.i., whereas no inflammatory foci could be assessed in other organs such as the heart or the large intestines (Liesenfeld et al., 1996).

In our present low-dose *T. gondii* infection study, parasitic DNA were detectable only in ileal *ex vivo* biopsies taken at day 10 p.i., but were below the detection limit in any other intestinal or extra-intestinal *ex vivo* biopsies taken during the observation period further underlining that the observed inflammatory changes are rather due to the immunological responses of the initial *T. gondii* challenge with one single cyst only. One needs to take into consideration that the (parasitic) pathogen does not necessarily need to be permanently in the infected body compartment in order to induce inflammatory sequelae. It is rather the initial hit set by the pathogen to the host subsequently tipping the balance toward immunopathology (Alutis et al., 2015; Heimesaat et al., 2016a,b,c; Grunau et al., 2017).

It is well known that the fine-tuned interactions between the complex intestinal bacterial ecosystem and the host are critical for immune homeostasis, cell physiology and resistance to morbidities (Ekmekciu et al., 2017a). Hence, disturbances in the orchestrated commensal gut microbiota composition are associated with the development and outcome of immunopathological diseases including gastrointestinal inflammation (Heimesaat et al., 2006; Erridge et al., 2010; Bereswill et al., 2011; Fiebiger et al., 2016; Ekmekciu et al., 2017a). Our culture-independent survey of the gut bacterial changes during low-dose *T. gondii* infection revealed that until day 10 p.i. (when the severity of induced ileitis was maximum) Gram-negative bacterial commensals such as enterobacteria including *E. coli* had increased in the inflamed ileal lumen, whereas conversely, Gram-positive lactobacilli and clostridia as well as *Mouse Intestinal Bacteroides* had decreased. These observations are well in line with results derived from our lethal high-dose *T. gondii* infection studies given that ileitis development was accompanied by dramatic shifts in the ileal microbiota compositions toward an overgrowth with enterobacterial species, whereas bacterial commensal diversity was reduced and particularly lactobacilli and clostridia were virtually undetectable (Heimesaat et al., 2006, 2007a; Erridge et al., 2010). *T. gondii* induced ileitis was further aggravated by Toll-like receptor (TLR)−4 dependent signaling of lipopolysaccharide derived from the cell walls of the overgrowing Gram-negative species (Heimesaat et al., 2007a; Erridge et al., 2010). During the chronic phase of low-dose *T. gondii* infection (i.e., at days 36 and 57 p.i.), mice harbored fewer bifidobacteria but also lactobacilli in the small intestines as compared to uninfected controls. Particularly bifidobacteria and lactobacilli are considered “health-beneficial” commensals with immuno-modulatory properties and sharing important functions during immune homeostasis (Bereswill et al., 2017; Ekmekciu et al., 2017b,c; Heimesaat et al., 2017). Dysbiosis with deprived intestinal bifidobacterial loads has been shown to be associated with celiac disease, irritable bowel syndrome, inflammatory bowel disease (IBD) and atopic diseases, for instance (Tojo et al., 2014). Furthermore, acute ileitis was more pronounced in high-dose *T. gondii* infected conventional mice that were gene deficient for either TLR-9 or nucleotide-binding oligomerization domain (NOD) 2 and were both lacking intestinal bifidobacterial commensals (Bereswill et al., 2014; Heimesaat et al., 2014).

The murine low-dose *T. gondii* infection model has most commonly been used in order to investigate parasitic inflammation of the central nervous system given that upon intraperitoneal challenge with <10 *T. gondii* cysts of the ME49 strain susceptible mice develop chronic cerebral inflammation (Dunay et al., 2010; Biswas et al., 2015; Mohle et al., 2016; Lang et al., 2018; Dusedau et al., 2019). However, for investigating subacute/chronic intestinal and extra-intestinal (besides neurological) immunopathological changes the low-dose infection model has only been applied in single studies so far (Dunay et al., 2008; Escher et al., 2018; Heimesaat et al., 2018a).

In conclusion, the here provided long-term kinetic survey of immunopathological sequalae in intestinal, extra-intestinal and systemic compartments following peroral low-dose *T. gondii* infection provides valuable corner stones for a better understanding of the complex interactions within the triangle relationship of (parasitic) pathogens, the host immunity and the commensal gut microbiota during subacute and chronic intestinal inflammation. Furthermore, the low-dose *T. gondii* infection model may be applied as valuable gut inflammation model in future pre-clinical studies in order to test potential treatment options for intestinal inflammatory conditions in humans (Bereswill et al., 2019).

## Ethics Statement

Following approval by the local authorities for animal experiments (für Gesundheit und Soziales, LaGeSo, Berlin, registration numbers G244/98), mouse experiments were performed in accordance with the European Guidelines for animal welfare (2010/63/EU). Clinical conditions of mice were assessed at least once daily.

## Author Contributions

MH designed and performed experiments, analyzed data, wrote paper. ID critically discussed experimental design and results, co-edited paper. SB provided advice in experimental design, critically discussed results, co-edited paper.

### Conflict of Interest Statement

The authors declare that the research was conducted in the absence of any commercial or financial relationships that could be construed as a potential conflict of interest.

## Supplementary Material

Figure S1 Representative photomicrographs illustrating inflammatory changes and distinct immune cell populations in the ileum over time following peroral low-dose *T. gondii* infection. Mice were perorally infected with one cyst of *T. gondii* on day 0 and surveyed at days (d) 10, 36, and 57 post-infection (p.i.). Naive (N) mice served as uninfected controls. Representative photomicrographs taken from the ileum illustrate **(A)** histopathological changes (H&E staining) and **(B)** apoptotic (caspase3+, Casp3+) cells, **(C)** proliferating (Ki67+) cells, **(D)** T lymphocytes (CD3+), and **(E)** B lymphocytes (B220+) in immunohistochemically stained paraffin sections at respective time points (100 x magnification, scale bar 100 μm).

Figure S2 Representative photomicrographs illustrating inflammatory changes and distinct immune cell populations in the colon over time following peroral low-dose *T. gondii* infection. Mice were perorally infected with one cyst of *T. gondii* on day 0 and surveyed at days (d) 10, 36, and 57 post-infection (p.i.). Naive (N) mice served as uninfected controls. Representative photomicrographs taken from the colon illustrate **(A)** histopathological changes (H&E staining) and **(B)** apoptotic (caspase3+, Casp3+) cells, **(C)** proliferating (Ki67+) cells, **(D)** T lymphocytes (CD3+), and **(E)** B lymphocytes (B220+) in immunohistochemically stained paraffin sections at respective time points (100 x magnification, scale bar 100 μm).

Figure S3 Representative photomicrographs illustrating inflammatory changes and distinct immune cell populations in the liver over time following peroral low-dose *T. gondii* infection. Mice were perorally infected with one cyst of *T. gondii* on day 0 and surveyed at days (d) 10, 36, and 57 post-infection (p.i.). Naive (N) mice served as uninfected controls. Representative photomicrographs taken from the liver illustrate **(A)** apoptotic (caspase3+, Casp3+) cells, **(B)** macrophages/monocytes (F4/80+), **(C)** T lymphocytes (CD3+), and **(D)** B lymphocytes (B220+) in immunohistochemically stained paraffin sections at respective time points (100 x magnification, scale bar 100 μm).

Figure S4 Representative photomicrographs illustrating inflammatory changes and distinct immune cell populations in the kidneys over time following peroral low-dose *T. gondii* infection. Mice were perorally infected with one cyst of *T. gondii* on day 0 and surveyed at days (d) 10, 36, and 57 post-infection (p.i.). Naive (N) mice served as uninfected controls. Representative photomicrographs taken from the kidneys illustrate **(A)** apoptotic (caspase3+, Casp3+) cells, **(B)** T lymphocytes (CD3+), and **(C)** B lymphocytes (B220+) in immunohistochemically stained paraffin sections at respective time points (100 x magnification, scale bar 100 μm).

Figure S5 Representative photomicrographs illustrating inflammatory changes and distinct immune cell populations in the lungs over time following peroral low-dose *T. gondii* infection. Mice were perorally infected with one cyst of *T. gondii* on day 0 and surveyed at days (d) 10, 36, and 57 post-infection (p.i.). Naive (N) mice served as uninfected controls. Representative photomicrographs taken from the lungs illustrate **(A)** apoptotic (caspase3+, Casp3+) cells, **(B)** macrophages/monocytes (F4/80+), **(C)** T lymphocytes (CD3+), and **(D)** B lymphocytes (B220+) in immunohistochemically stained paraffin sections at respective time points (100 x magnification, scale bar 100 μm).

Figure S6 Microscopic inflammatory sequelae in the heart muscle over time following peroral low-dose *T. gondii* infection. Mice were perorally infected with one cyst of *T. gondii* on day 0 and surveyed at days (d) 10, 36, and 57 post-infection (p.i.; closed circles). Naive (N) mice served as uninfected controls (open circles). Out of six representative high power fields (HPF, 400x magnification) per animal the average numbers of **(A)** apoptotic (casapse3+, Casp3+) cells, of **(B)** T lymphocytes (CD3+) and **(C)** B lymphocytes were assessed in immunohistochemically stained cardiac paraffin sections. Medians (black bars), levels of significance (*p*-values) as determined by one-way ANOVA test followed by Tukey post-correction for multiple comparisons and numbers of analyzed mice (in parentheses) are indicated. Data were pooled from four independent experiments.

Figure S7 Representative photomicrographs illustrating inflammatory changes and distinct immune cell populations in the heart over time following peroral low-dose *T. gondii* infection. Mice were perorally infected with one cyst of *T. gondii* on day 0 and surveyed at days (d) 10, 36, and 57 post-infection (p.i.). Naive (N) mice served as uninfected controls. Representative photomicrographs taken from the heart illustrate **(A)** apoptotic (caspase3+, Casp3+) cells, **(B)** T lymphocytes (CD3+), and **(C)** B lymphocytes (B220+) in immunohistochemically stained paraffin sections at respective time points (100 x magnification, scale bar 100 μm).

## Abbreviations

CBA: cytometric bead array
H&E: hematoxylin and eosin
IBD: inflammatory bowel disease
IFN: interferon
IL: interleukin
MLN: mesenteric lymph nodes
NOD: nucleotide-oligomerization-domain
PBS: phosphate-buffered saline
p.i.: post-infection
SPF: specific pathogen free
TLR: toll-like receptor
TNF: tumor necrosis factor
WT: wildtype.

## References

[B1] Alutis M. E. Grundmann U. Fischer A. Hagen U. Kuhl A. A. Gobel U. B.. (2015). The role of gelatinases in *Campylobacter jejuni* infection of gnotobiotic mice. Eur. J. Microbiol. Immunol. 5, 256–267. 10.1556/1886.2015.00033 26716014 PMC4681353

[B2] Bereswill S. Ekmekciu I. Escher U. Fiebiger U. Stingl K. Heimesaat M. M. (2017). Lactobacillus johnsonii ameliorates intestinal, extra-intestinal and systemic pro-inflammatory immune responses following murine *Campylobacter jejuni* infection. Sci. Rep. 7:2138. 10.1038/s41598-017-02436-2 28522817 PMC5437126

[B3] Bereswill S. Escher U. Grunau A. Kühl A. A. Dunay I. R. Tamas A. (2019). Pituitary adenylate cyclase-activating polypeptide - a novel treatment option for subacute ileitis in mice harboring a human gut microbiota. Front. Immunol. 10 10.3389/fimmu.2019.00554 PMC6438926 30967875

[B4] Bereswill S. Fischer A. Plickert R. Haag L. M. Otto B. Kuhl A. A.. (2011). Novel murine infection models provide deep insights into the “menage a trois” of *Campylobacter jejuni*, microbiota and host innate immunity. PLoS ONE 6:e20953. 10.1371/annotation/5247af81-4595-44b7-9c3f-2e45ad85abfa 21698299 PMC3115961

[B5] Bereswill S. Kuhl A. A. Alutis M. Fischer A. Mohle L. Struck D.. (2014). The impact of toll-like-receptor-9 on intestinal microbiota composition and extra-intestinal sequelae in experimental *Toxoplasma gondii* induced ileitis. Gut Pathog. 6:19. 10.1186/1757-4749-6-19 24932221 PMC4057803

[B6] Biswas A. Bruder D. Wolf S. A. Jeron A. Mack M. Heimesaat M. M.. (2015). Ly6C(high) monocytes control cerebral toxoplasmosis. J. Immunol. 194, 3223–3235. 10.4049/jimmunol.1402037 25710908

[B7] Derouin F. Garin Y. J. (1991). *Toxoplasma gondii*: blood and tissue kinetics during acute and chronic infections in mice. Exp. Parasitol. 73, 460–468. 10.1016/0014-4894(91)90070-D 1959573

[B8] Dubey J. P. (1997). Bradyzoite-induced murine toxoplasmosis: stage conversion, pathogenesis, and tissue cyst formation in mice fed bradyzoites of different strains of *Toxoplasma gondii*. J. Eukaryot. Microbiol. 44, 592–602. 10.1111/j.1550-7408.1997.tb05965.x 9435131

[B9] Dunay I. R. Damatta R. A. Fux B. Presti R. Greco S. Colonna M.. (2008). Gr1(+) inflammatory monocytes are required for mucosal resistance to the pathogen *Toxoplasma gondii*. Immunity 29, 306–317. 10.1016/j.immuni.2008.05.019 18691912 PMC2605393

[B10] Dunay I. R. Fuchs A. Sibley L. D. (2010). Inflammatory monocytes but not neutrophils are necessary to control infection with *Toxoplasma gondii* in mice. Infect. Immun. 78, 1564–1570. 10.1128/IAI.00472-09 20145099 PMC2849397

[B11] Dusedau H. P. Kleveman J. Figueiredo C. A. Biswas A. Steffen J. Kliche S. (2019). p75(NTR) regulates brain mononuclear cell function and neuronal structure in toxoplasma infection-induced neuroinflammation. Glia 67, 193–211. 10.1002/glia.23553 30597659 PMC6590406

[B12] Ekmekciu I. von Klitzing E. Fiebiger U. Escher U. Neumann C. Bacher P.. (2017a). Immune responses to broad-spectrum antibiotic treatment and fecal microbiota transplantation in mice. Front. Immunol. 8:397. 10.3389/fimmu.2017.00397 28469619 PMC5395657

[B13] Ekmekciu I. von Klitzing E. Fiebiger U. Neumann C. Bacher P. Scheffold A. (2017b). The probiotic compound VSL#3 modulates mucosal, peripheral, and systemic immunity following murine broad-spectrum antibiotic treatment. Front. Cell. Infect. Microbiol. 7:167 10.3389/fcimb.2017.00167 28529928 PMC5418240

[B14] Ekmekciu I. von Klitzing E. Neumann C. Bacher P. Scheffold A. Bereswill S.. (2017c). Fecal microbiota transplantation, commensal *Escherichia coli* and lactobacillus johnsonii strains differentially restore intestinal and systemic adaptive immune cell populations following broad-spectrum antibiotic treatment. Front. Microbiol. 8:2430. 10.3389/fmicb.2017.02430 29321764 PMC5732213

[B15] Erridge C. Duncan S. H. Bereswill S. Heimesaat M. M. (2010). The induction of colitis and ileitis in mice is associated with marked increases in intestinal concentrations of stimulants of TLRs 2, 4, and 5. PLoS ONE 5:e9125. 10.1371/journal.pone.0009125 20161736 PMC2817728

[B16] Escher U. Giladi E. Dunay I. R. Bereswill S. Gozes I. Heimesaat M. M. (2018). Anti-inflammatory effects of the octapeptide NAP in human microbiota-associated mice suffering from subacute ileitis. Eur. J. Microbiol. Immunol. 8, 34–40. 10.1556/1886.2018.00006 29997909 PMC6038539

[B17] Fiebiger U. Bereswill S. Heimesaat M. M. (2016). Dissecting the interplay between intestinal microbiota and host immunity in health and disease: lessons learned from germfree and gnotobiotic animal models. Eur. J. Microbiol. Immunol. 6, 253–271. 10.1556/1886.2016.00036 27980855 PMC5146645

[B18] Grunau A. Escher U. Kuhl A. A. Bereswill S. Heimesaat M. M. (2017). Toll-like receptor-4 differentially mediates intestinal and extra-intestinal immune responses upon multi-drug resistant *Pseudomonas aeruginosa* association of IL10(-/-) mice with chronic colitis. Gut Pathog. 9:61. 10.1186/s13099-017-0211-z 29151895 PMC5678768

[B19] Heimesaat M. M. Alutis M. E. Grundmann U. Fischer A. Gobel U. B. Bereswill S. (2016c). The role of IL-23, IL-22, and IL-18 in *Campylobacter jejuni* infection of conventional infant mice. Eur. J. Microbiol. Immunol. 6, 124–136. 10.1556/1886.2016.00008 27429795 PMC4936335

[B20] Heimesaat M. M. Bereswill S. Fischer A. Fuchs D. Struck D. Niebergall J.. (2006). Gram-negative bacteria aggravate murine small intestinal Th1-type immunopathology following oral infection with *Toxoplasma gondii*. J. Immunol. 177, 8785–8795. 10.4049/jimmunol.177.12.8785 17142781

[B21] Heimesaat M. M. Dunay I. R. Alutis M. Fischer A. Mohle L. Gobel U. B.. (2014). Nucleotide-oligomerization-domain-2 affects commensal gut microbiota composition and intracerebral immunopathology in acute *Toxoplasma gondii* induced murine ileitis. PLoS ONE 9:e105120. 10.1371/journal.pone.0105120 25141224 PMC4139296

[B22] Heimesaat M. M. Escher U. Grunau A. Fiebiger U. Bereswill S. (2018a). Peroral low-dose *Toxoplasma gondii* infection of human microbiota-associated mice - a subacute ileitis model to unravel pathogen-host interactions. Eur. J. Microbiol. Immunol. 8, 53–61. 10.1556/1886.2018.00005 29997912 PMC6038537

[B23] Heimesaat M. M. Fischer A. Jahn H. K. Niebergall J. Freudenberg M. Blaut M.. (2007a). Exacerbation of murine ileitis by toll-like receptor 4 mediated sensing of lipopolysaccharide from commensal *Escherichia coli*. Gut 56, 941–948. 10.1136/gut.2006.104497 17255219 PMC1994376

[B24] Heimesaat M. M. Fischer A. Siegmund B. Kupz A. Niebergall J. Fuchs D.. (2007b). Shift towards pro-inflammatory intestinal bacteria aggravates acute murine colitis via toll-like receptors 2 and 4. PLoS ONE 2:e662. 10.1371/journal.pone.0000662 17653282 PMC1914380

[B25] Heimesaat M. M. Giladi E. Kuhl A. A. Bereswill S. Gozes I. (2018b). The octapetide NAP alleviates intestinal and extra-intestinal anti-inflammatory sequelae of acute experimental colitis. Peptides 101:1–9. 10.1016/j.peptides.2017.12.023 29288684

[B26] Heimesaat M. M. Grundmann U. Alutis M. E. Fischer A. Gobel U. B. Bereswill S. (2016a). The IL-23/IL-22/IL-18 axis in murine *Campylobacter jejuni* infection. Gut Pathog. 8:21. 10.1186/s13099-016-0106-4 27385977 PMC4934010

[B27] Heimesaat M. M. Grundmann U. Alutis M. E. Fischer A. Gobel U. B. Bereswill S. (2016b). Colonic expression of genes encoding inflammatory mediators and gelatinases during *Campylobacter Jejuni* infection of conventional infant mice. Eur. J. Microbiol. Immunol. 6, 137–146. 10.1556/1886.2016.00009 27429796 PMC4936336

[B28] Heimesaat M. M. Nogai A. Bereswill S. Plickert R. Fischer A. Loddenkemper C.. (2010). MyD88/TLR9 mediated immunopathology and gut microbiota dynamics in a novel murine model of intestinal graft-versus-host disease. Gut 59, 1079–1087. 10.1136/gut.2009.197434 20639251

[B29] Heimesaat M. M. Reifenberger G. Vicena V. Illes A. Horvath G. Tamas A.. (2017). Intestinal microbiota changes in mice lacking pituitary adenylate cyclase activating polypeptide (PACAP) - bifidobacteria make the difference. Eur. J. Microbiol. Immunol. 7, 187–199. 10.1556/1886.2017.00021 29034108 PMC5632746

[B30] Jankovic D. Kugler D. G. Sher A. (2010). IL-10 production by CD4+ effector T cells: a mechanism for self-regulation. Mucosal Immunol. 3, 239–246. 10.1038/mi.2010.8 20200511 PMC4105209

[B31] Lang D. Schott B. H. van Ham M. Morton L. Kulikovskaja L. Herrera-Molina R.. (2018). Chronic toxoplasma infection is associated with distinct alterations in the synaptic protein composition. J. Neuroinflammation 15, 216. 10.1186/s12974-018-1242-1 30068357 PMC6090988

[B32] Liesenfeld O. (2002). Oral infection of C57BL/6 mice with *Toxoplasma gondii*: a new model of inflammatory bowel disease?. J. Infect. Dis. 185(Suppl 1), S96–101. 10.1086/338006 11865446

[B33] Liesenfeld O. Kang H. Park D. Nguyen T. A. Parkhe C. V. Watanabe H.. (1999). TNF-alpha, nitric oxide and IFN-gamma are all critical for development of necrosis in the small intestine and early mortality in genetically susceptible mice infected perorally with *Toxoplasma gondii*. Parasite Immunol. 21, 365–376. 10.1046/j.1365-3024.1999.00237.x 10417671

[B34] Liesenfeld O. Kosek J. Remington J. S. Suzuki Y. (1996). Association of CD4+ T cell-dependent, interferon-gamma-mediated necrosis of the small intestine with genetic susceptibility of mice to peroral infection with *Toxoplasma gondii*. J. Exp. Med. 184, 597–607. 10.1084/jem.184.2.597 8760813 PMC2192709

[B35] McLeod R. Eisenhauer P. Mack D. Brown C. Filice G. Spitalny G. (1989a). Immune responses associated with early survival after peroral infection with *Toxoplasma gondii*. J. Immunol. 142, 3247–3255. 2496163

[B36] McLeod R. Skamene E. Brown C. R. Eisenhauer P. B. Mack D. G. (1989b). Genetic regulation of early survival and cyst number after peroral *Toxoplasma gondii* infection of A x B/B x A recombinant inbred and B10 congenic mice. J. Immunol. 143, 3031–3034. 2809214

[B37] Mohle L. Israel N. Paarmann K. Krohn M. Pietkiewicz S. Muller A.. (2016). Chronic *Toxoplasma gondii* infection enhances beta-amyloid phagocytosis and clearance by recruited monocytes. Acta Neuropathol. Commun. 4:25. 10.1186/s40478-016-0293-8 26984535 PMC4793516

[B38] Munoz M. Eidenschenk C. Ota N. Wong K. Lohmann U. Kuhl A. A.. (2015). Interleukin-22 induces interleukin-18 expression from epithelial cells during intestinal infection. Immunity 42, 321–331. 10.1016/j.immuni.2015.01.011 25680273

[B39] Munoz M. Heimesaat M. M. Danker K. Struck D. Lohmann U. Plickert R.. (2009). Interleukin (IL)-23 mediates *Toxoplasma gondii*-induced immunopathology in the gut via matrixmetalloproteinase-2 and IL-22 but independent of IL-17. J. Exp. Med. 206, 3047–3059. 10.1084/jem.20090900 19995958 PMC2806449

[B40] Munoz M. Liesenfeld O. Heimesaat M. M. (2011). Immunology of *Toxoplasma gondii*. Immunol. Rev. 240, 269–285. 10.1111/j.1600-065X.2010.00992.x 21349099

[B41] Rausch S. Held J. Fischer A. Heimesaat M. M. Kuhl A. A. Bereswill S.. (2013). Small intestinal nematode infection of mice is associated with increased enterobacterial loads alongside the intestinal tract. PLoS ONE 8:e74026. 10.1371/journal.pone.0074026 24040152 PMC3769368

[B42] Sumyuen M. H. Garin Y. J. Derouin F. (1995). Early kinetics of *Toxoplasma gondii* infection in mice infected orally with cysts of an avirulent strain. J. Parasitol. 81, 327–329. 10.2307/3283948 7707221

[B43] Suzuki Y. Sher A. Yap G. Park D. Neyer L. E. Liesenfeld O.. (2000). IL-10 is required for prevention of necrosis in the small intestine and mortality in both genetically resistant BALB/c and susceptible C57BL/6 mice following peroral infection with *Toxoplasma gondii*. J. Immunol. 164, 5375–5382. 10.4049/jimmunol.164.10.5375 10799901

[B44] Tojo R. Suarez A. Clemente M. G. de los Reyes-Gavilan C. G. Margolles A. Gueimonde M.. (2014). Intestinal microbiota in health and disease: role of bifidobacteria in gut homeostasis. World J. Gastroenterol. 20, 15163–15176. 10.3748/wjg.v20.i41.15163 25386066 PMC4223251

[B45] von Klitzing E. Ekmekciu I. Bereswill S. Heimesaat M. M. (2017a). Acute ileitis facilitates infection with multidrug resistant *Pseudomonas aeruginosa* in human microbiota-associated mice. Gut Pathog. 9:4. 10.1186/s13099-017-0154-4 28115993 PMC5241993

[B46] von Klitzing E. Ekmekciu I. Bereswill S. Heimesaat M. M. (2017c). Intestinal and systemic immune responses upon multi-drug resistant *Pseudomonas aeruginosa* colonization of mice harboring a human gut microbiota. Front. Microbiol. 8:2590. 10.3389/fmicb.2017.02590 29312263 PMC5744425

[B47] von Klitzing E. Ekmekciu I. Kuhl A. A. Bereswill S. Heimesaat M. M. (2017b). Intestinal, extra-intestinal and systemic sequelae of *Toxoplasma gondii* induced acute ileitis in mice harboring a human gut microbiota. PLoS ONE 12:e0176144. 10.1371/journal.pone.0176144 28414794 PMC5393883

[B48] von Klitzing E. Oz F. Ekmekciu I. Escher U. Bereswill S. Heimesaat M. M. (2017d). Comprehensive survey of intestinal microbiota changes in offspring of human microbiota-associated mice. Eur. J. Microbiol. Immunol. 7, 65–75. 10.1556/1886.2017.00002 28386472 PMC5372482

[B49] Vossenkamper A. Struck D. Alvarado-Esquivel C. Went T. Takeda K. Akira S.. (2004). Both IL-12 and IL-18 contribute to small intestinal Th1-type immunopathology following oral infection with *Toxoplasma gondii*, but IL-12 is dominant over IL-18 in parasite control. Eur. J. Immunol. 34, 3197–3207. 10.1002/eji.200424993 15368276

