# Supplementary material for: Comprehensive Kinetic Survey of Intestinal, Extra-Intestinal and Systemic Sequelae of Murine Ileitis Following Peroral Low-Dose Toxoplasma gondii Infection
Source: Front Cell Infect Microbiol. 2019 Apr 12;9:98. doi: 10.3389/fcimb.2019.00098 (PMC6474322; doi:10.3389/fcimb.2019.00098)

A

Apoptotic Cells (Casp3+) - LIVER

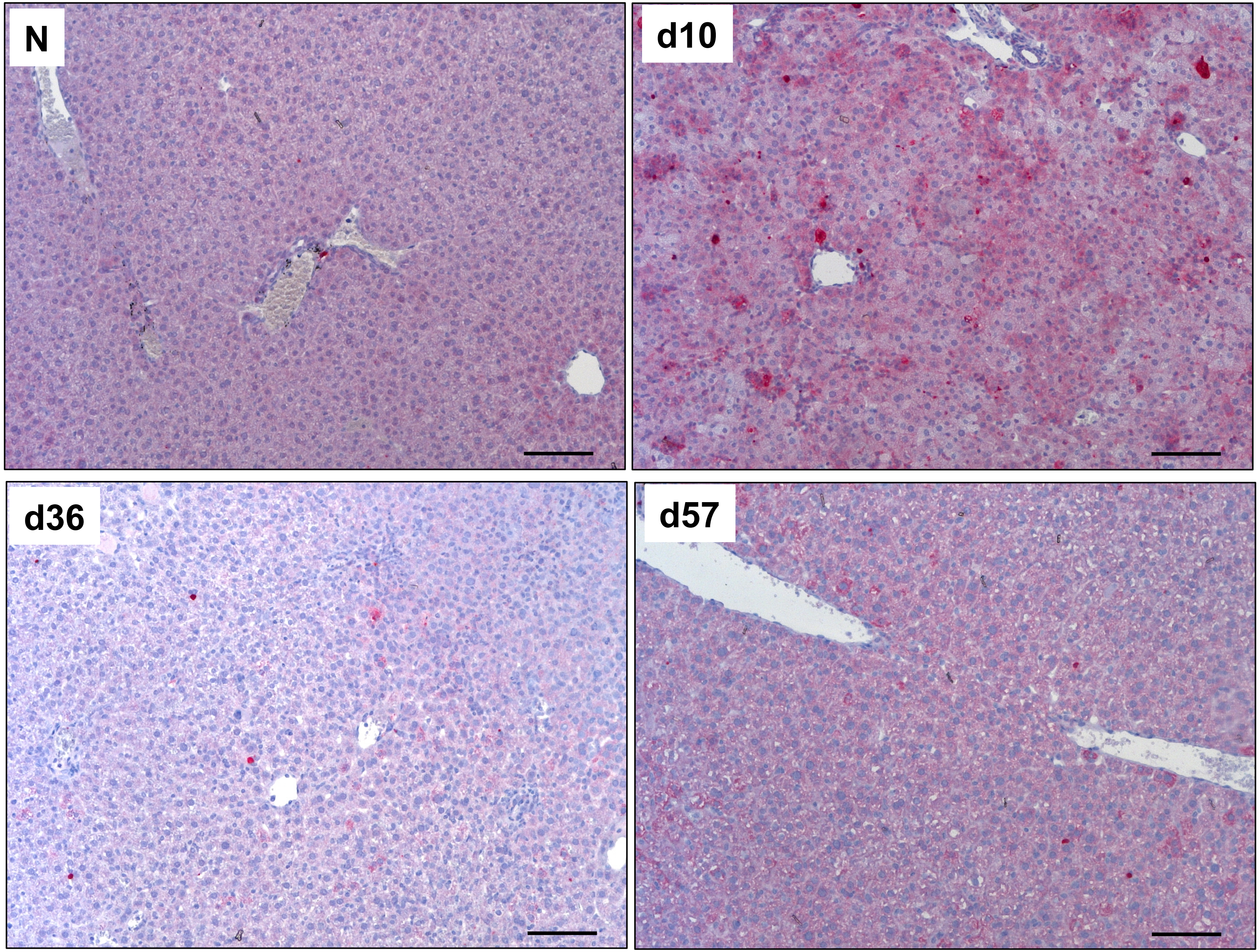

B

Macrophages / Monocytes (F4/80+) - LIVER

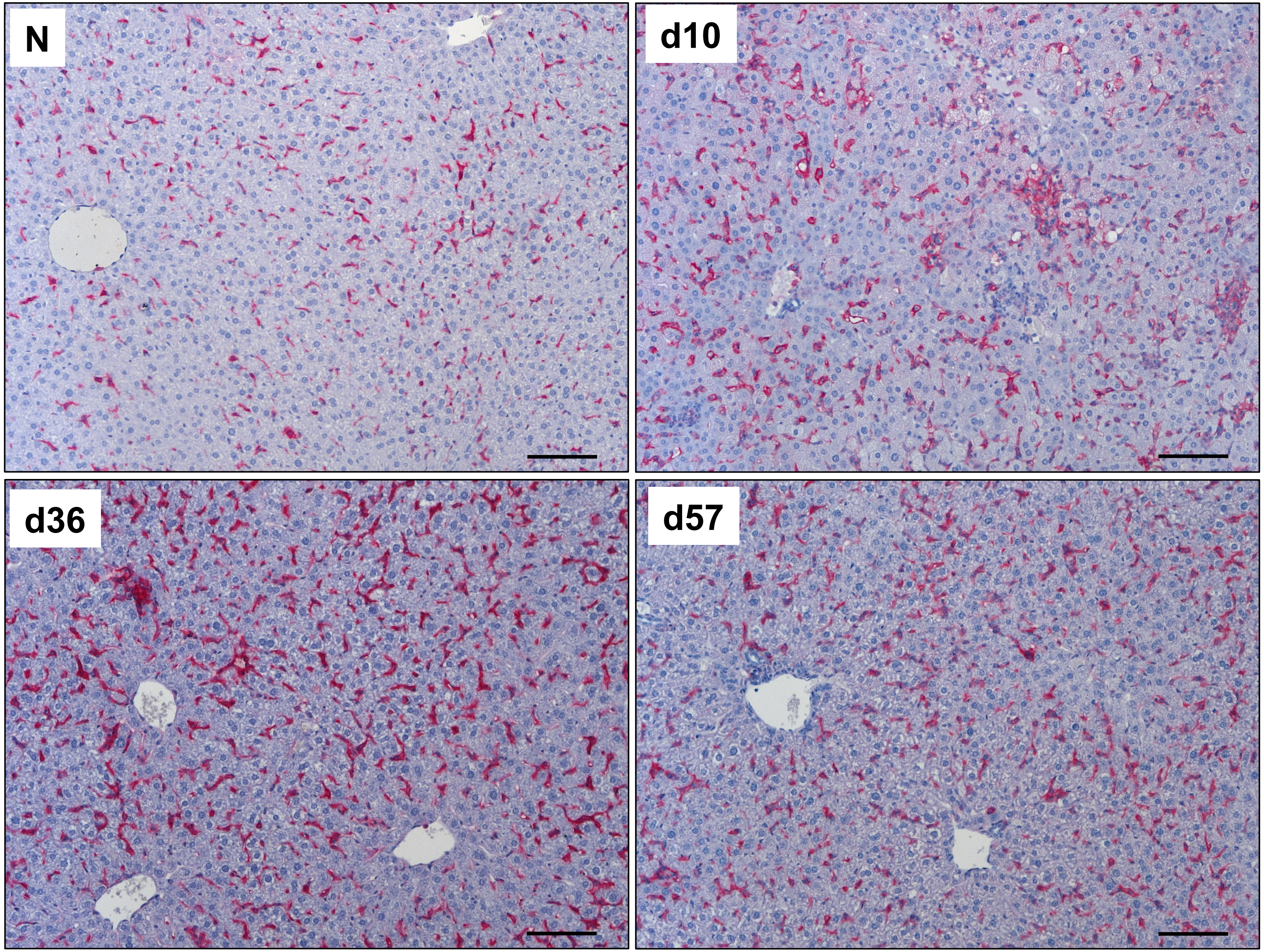

C

T Lymphocytes (CD3+) - LIVER

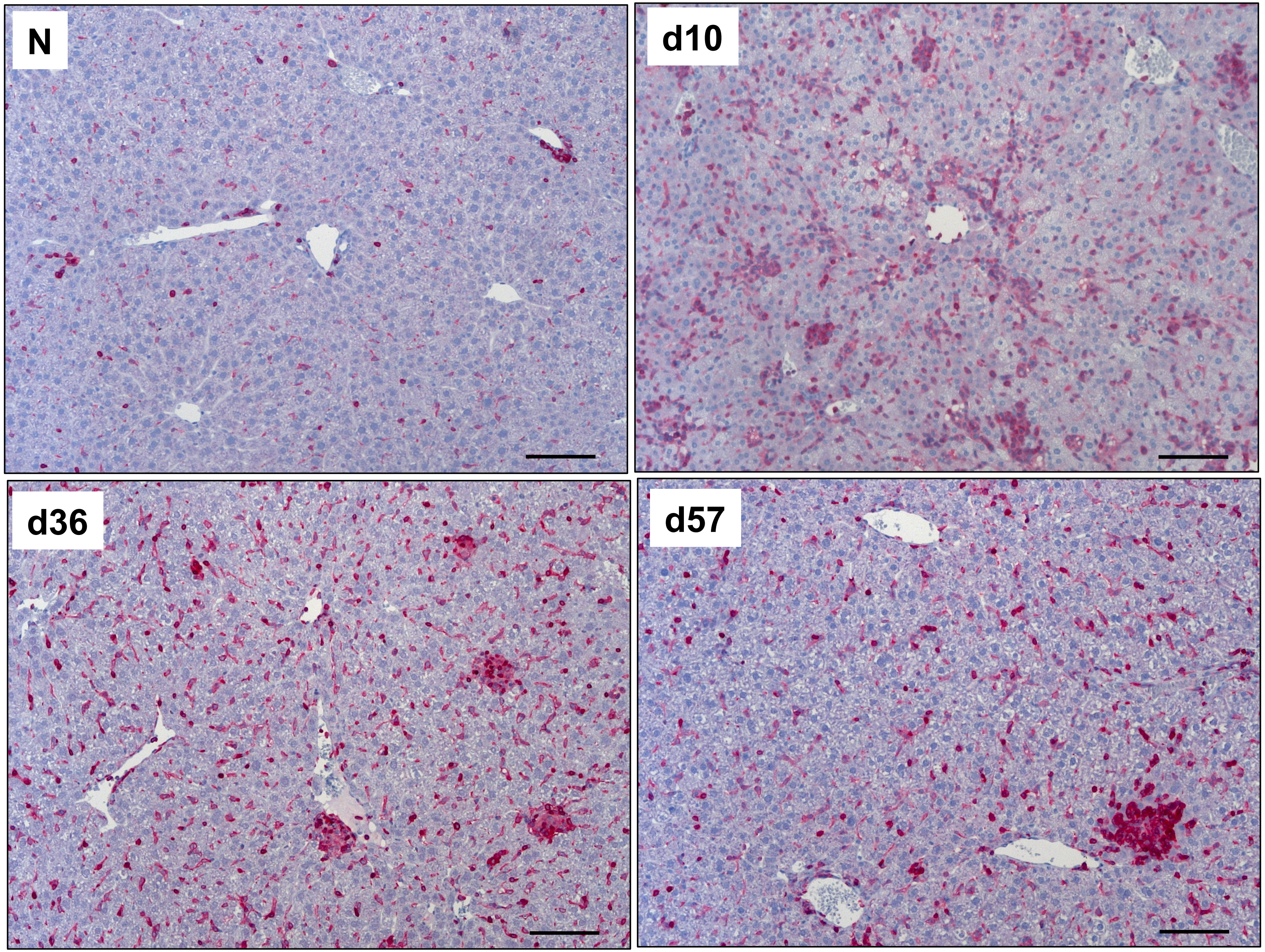

D

B Lymphocytes (B220+) - LIVER

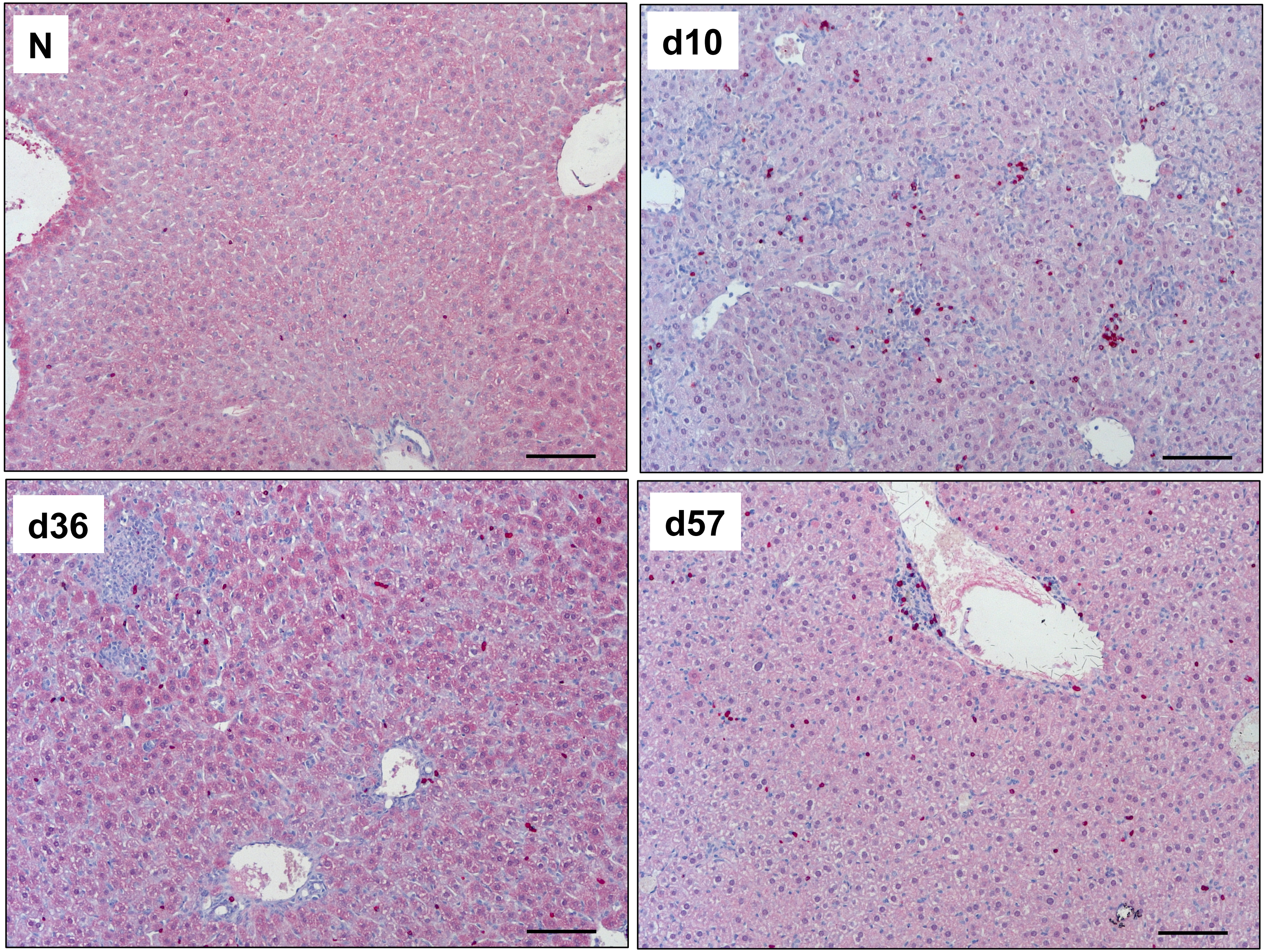

Supplement: Figure S3 — Representative photomicrographs illustrating inflammatory changes and distinct immune cell populations in the liver over time following peroral low-dose T. gondii infection. Mice were perorally infected with one cyst of T. gondii on day 0 and surveyed at days (d) 10, 36, and 57 post-infection (p.i.). Naive (N) mice served as uninfected controls. Representative photomicrographs taken from the liver illustrate (A) apoptotic (caspase3+, Casp3+) cells, (B) macrophages/monocytes (F4/80+), (C) T lymphocytes (CD3+), and (D) B lymphocytes (B220+) in immunohistochemically stained paraffin sections at respective time points (100 x magnification, scale bar 100 μm). [file Data_Sheet_3.PDF]
