# Supplementary figures and images for: Comprehensive Kinetic Survey of Intestinal, Extra-Intestinal and Systemic Sequelae of Murine Ileitis Following Peroral Low-Dose Toxoplasma gondii Infection
Source: Front Cell Infect Microbiol. 2019 Apr 12;9:98. doi: 10.3389/fcimb.2019.00098 (PMC6474322; doi:10.3389/fcimb.2019.00098)

**A**

# Apoptotic Cells (Casp3+) - KIDNEY

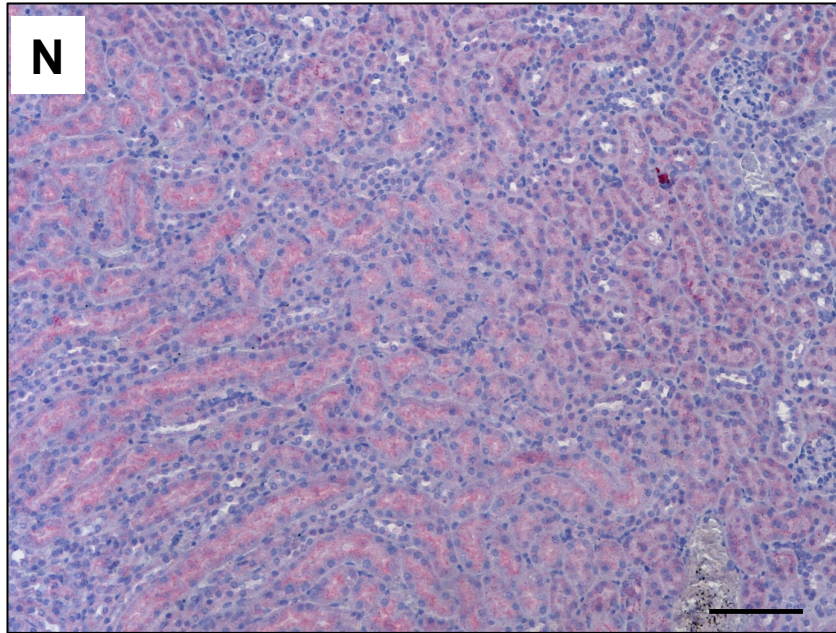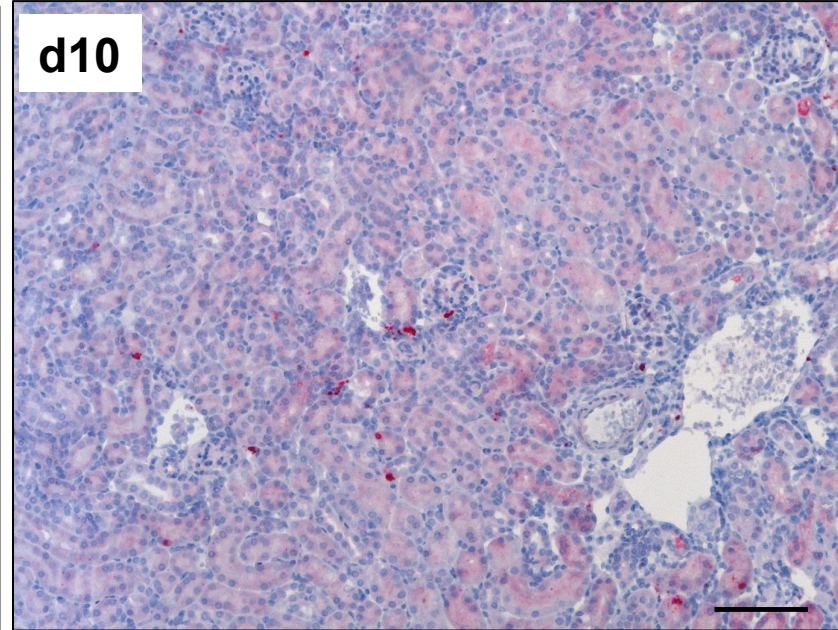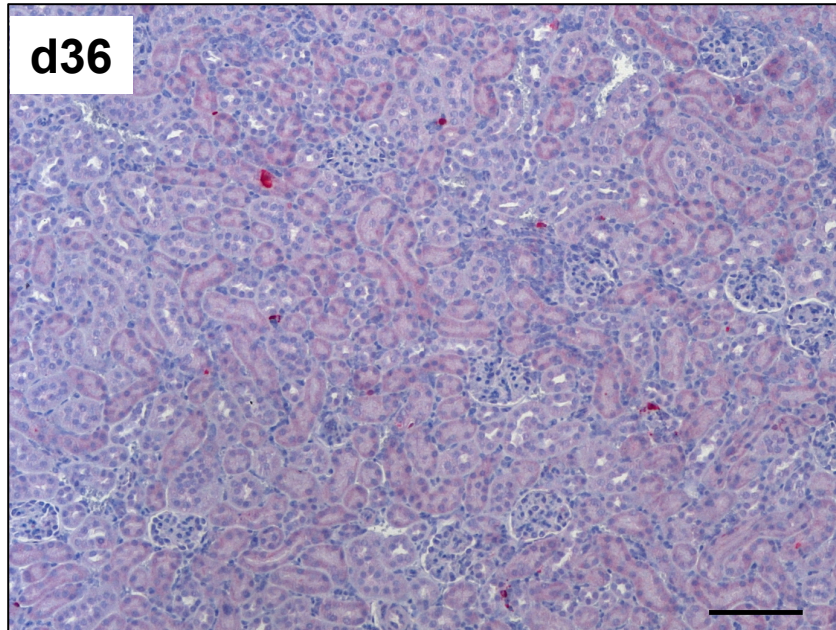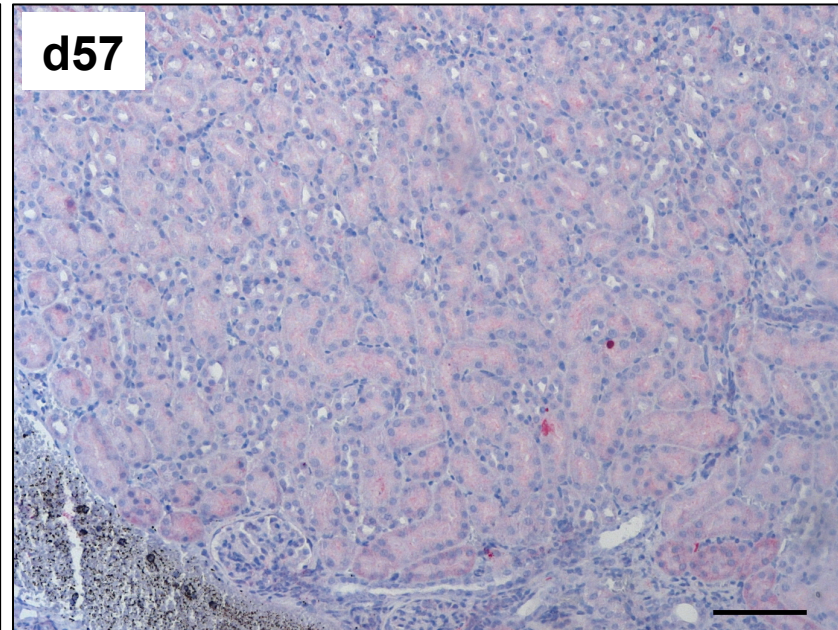

**B**

# T Lymphocytes (CD3+) - KIDNEY

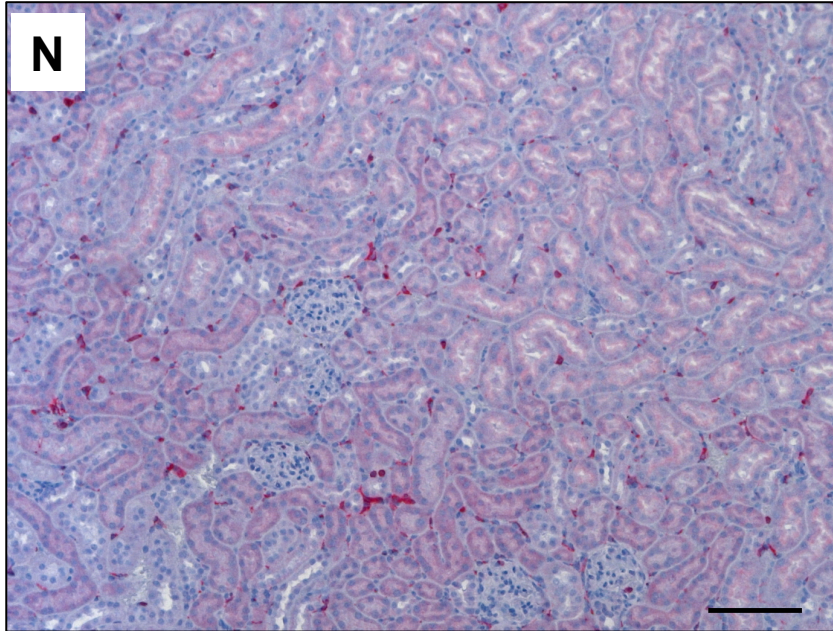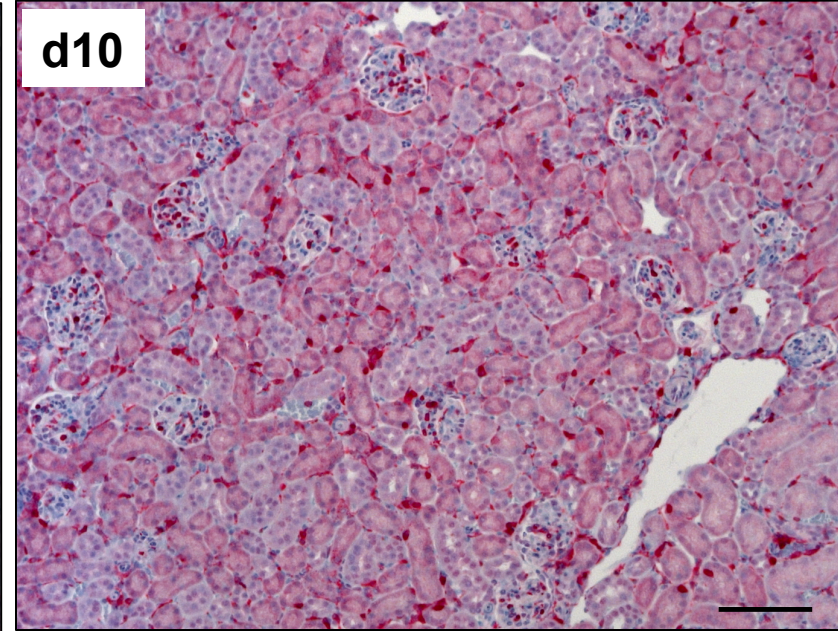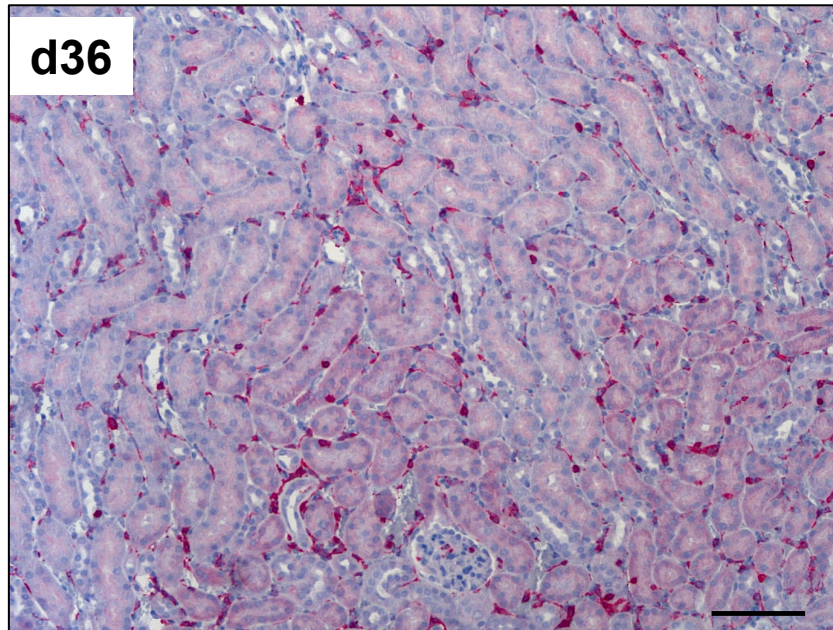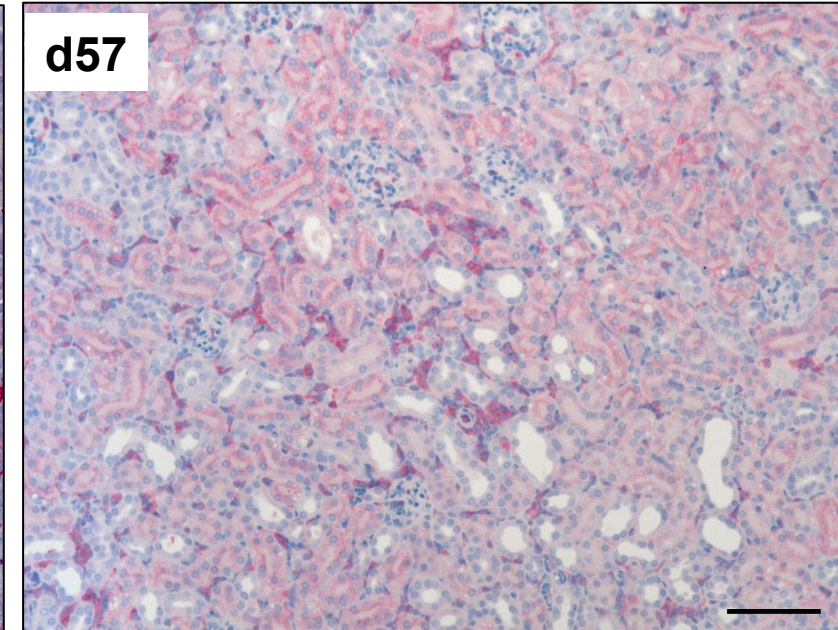

**C**

## **B Lymphocytes (B220+) - KIDNEY**

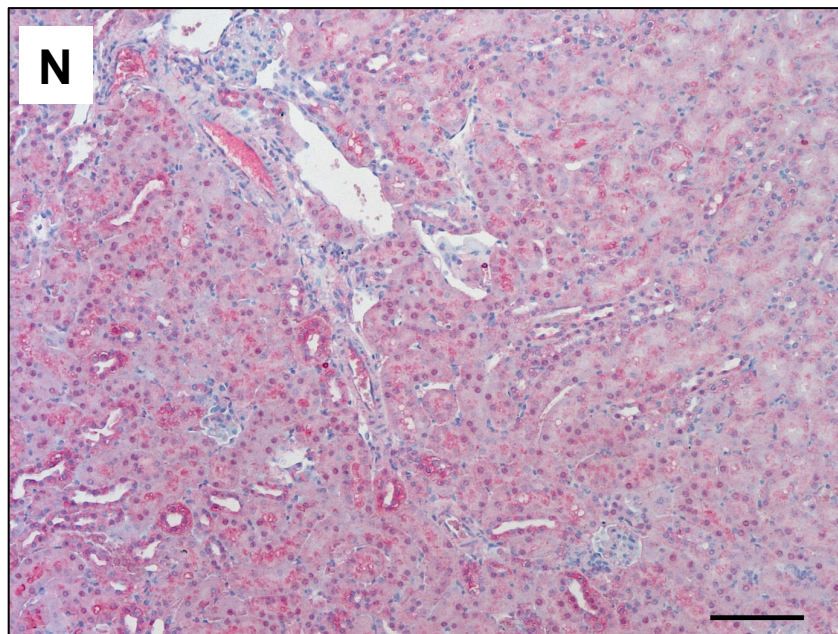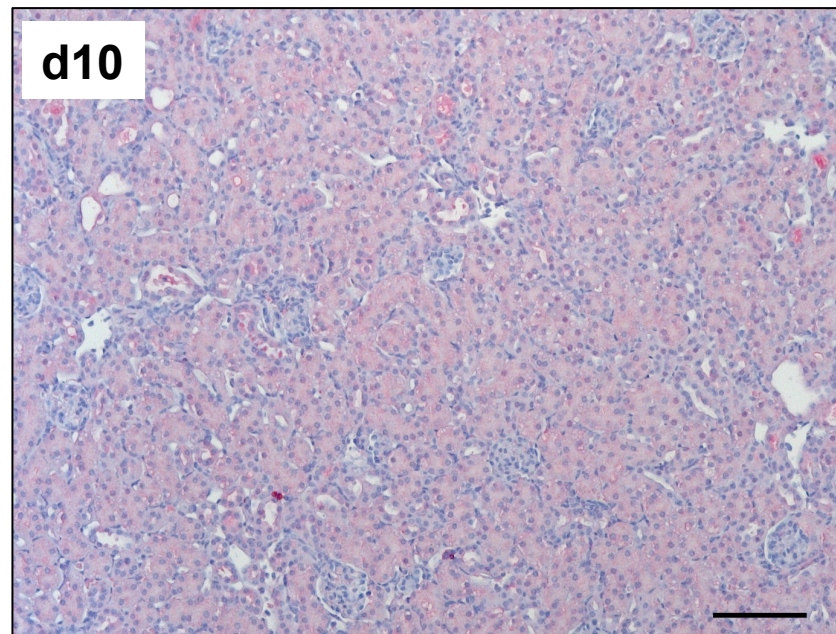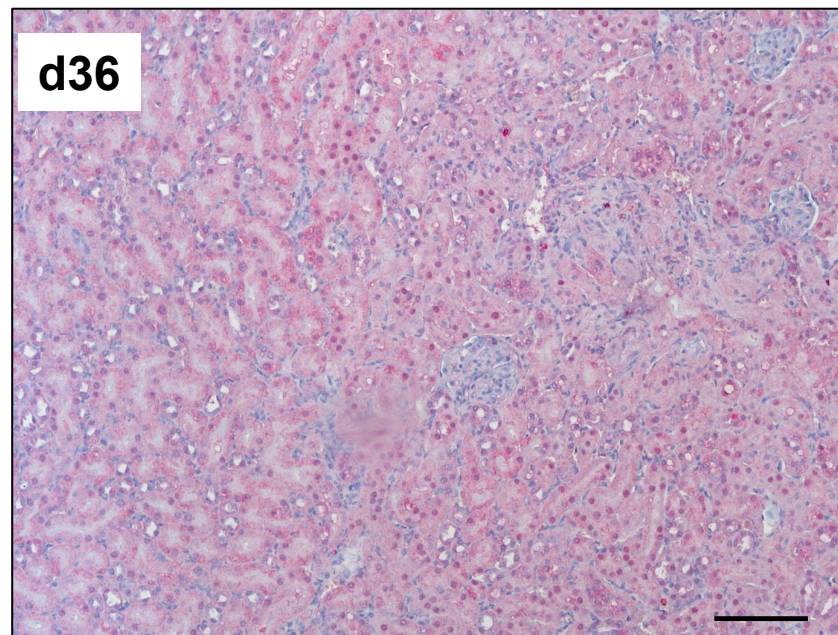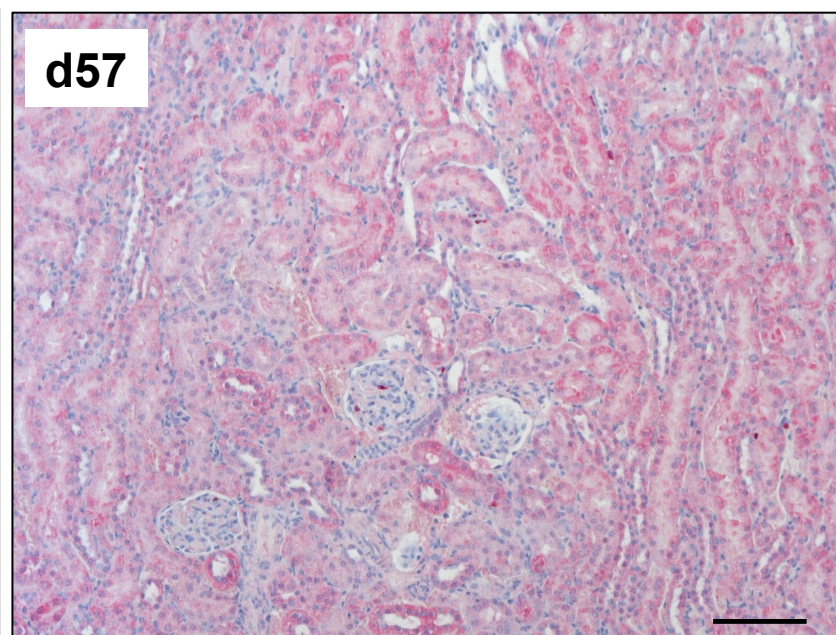

Supplement: Figure S4 — Representative photomicrographs illustrating inflammatory changes and distinct immune cell populations in the kidneys over time following peroral low-dose T. gondii infection. Mice were perorally infected with one cyst of T. gondii on day 0 and surveyed at days (d) 10, 36, and 57 post-infection (p.i.). Naive (N) mice served as uninfected controls. Representative photomicrographs taken from the kidneys illustrate (A) apoptotic (caspase3+, Casp3+) cells, (B) T lymphocytes (CD3+), and (C) B lymphocytes (B220+) in immunohistochemically stained paraffin sections at respective time points (100 x magnification, scale bar 100 μm). [file Data_Sheet_4.PDF]

# A

## Apoptotic Cells (Casp3+) - HEART

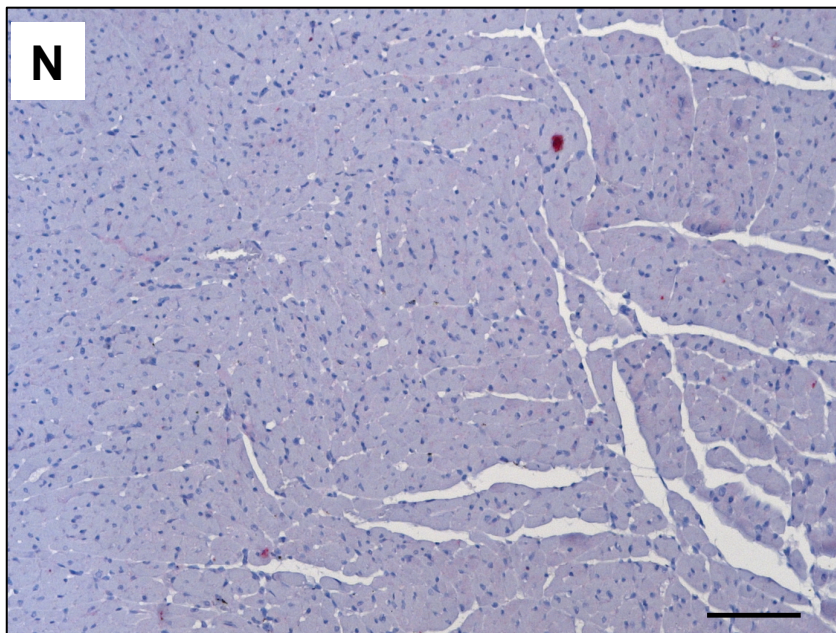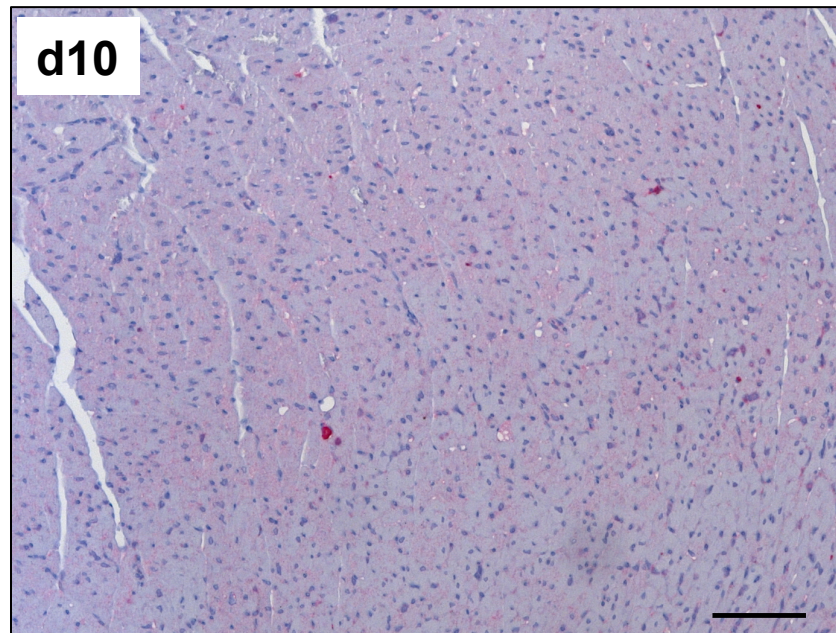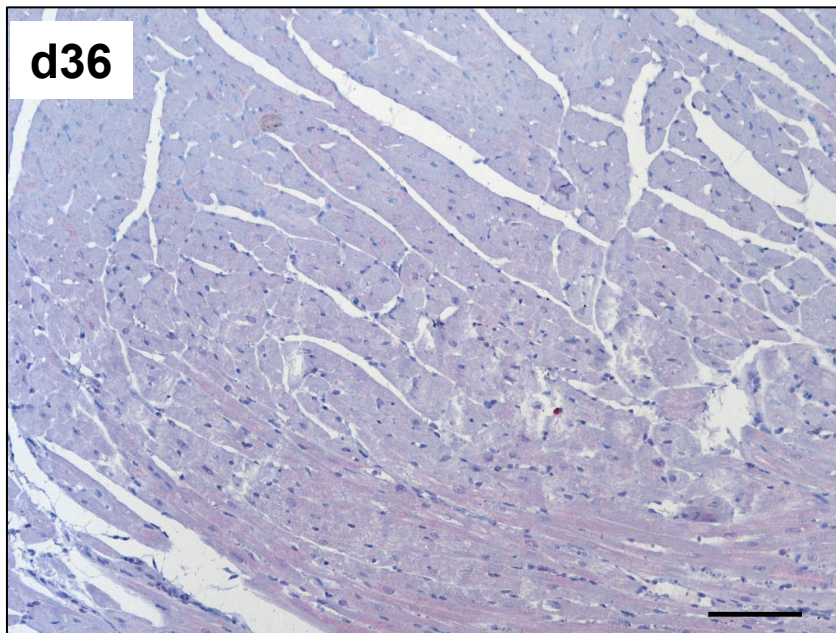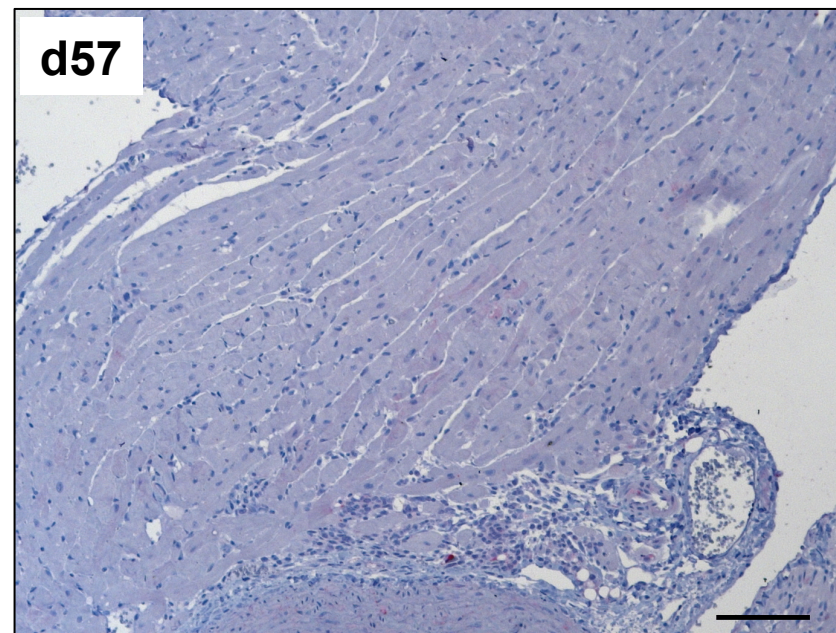

**B**

# T Lymphocytes (CD3+) - HEART

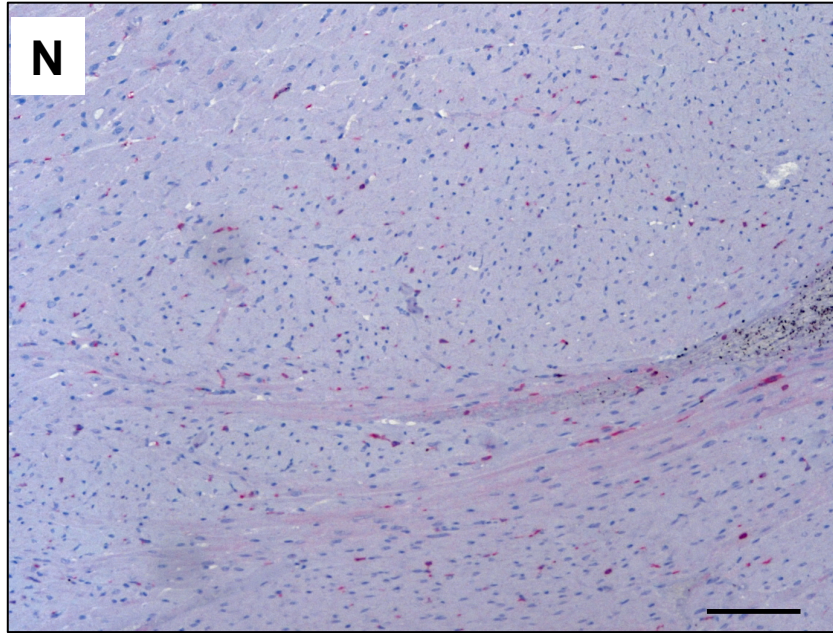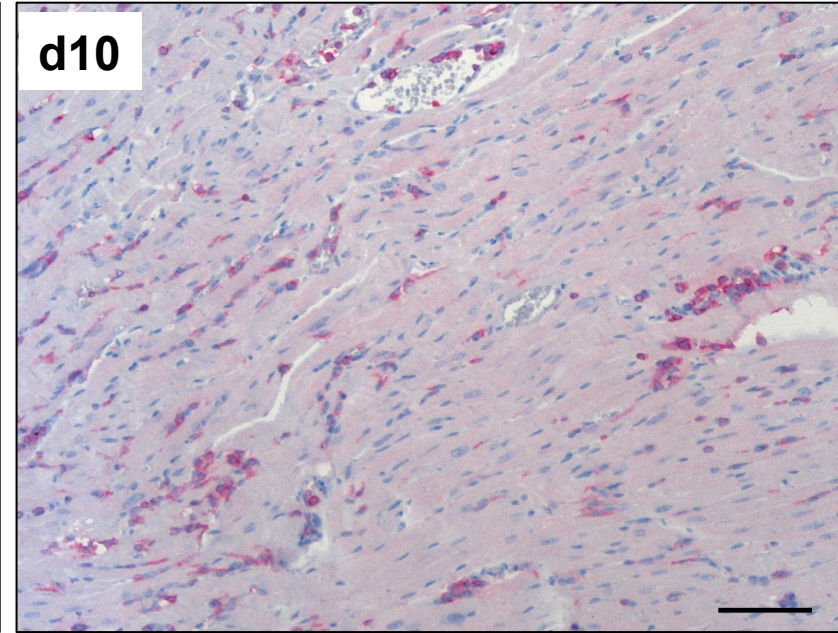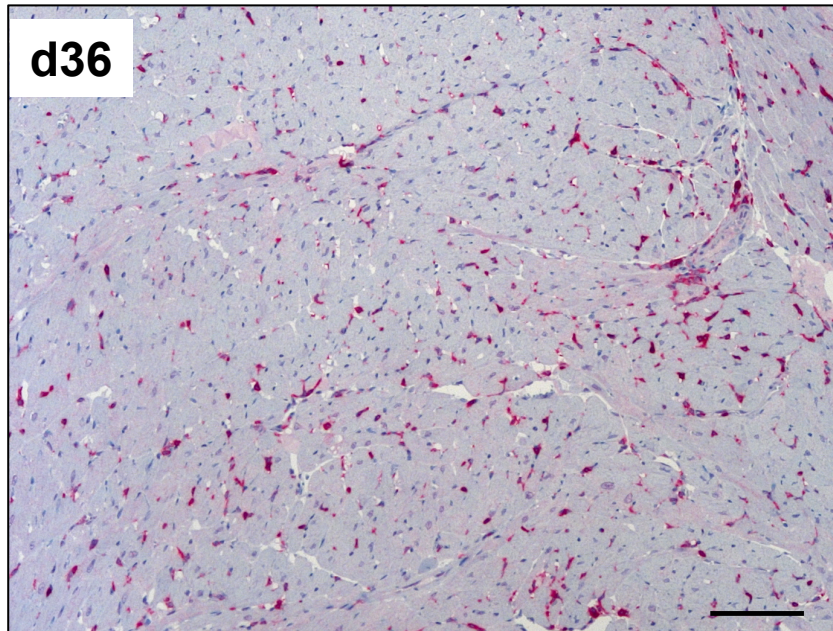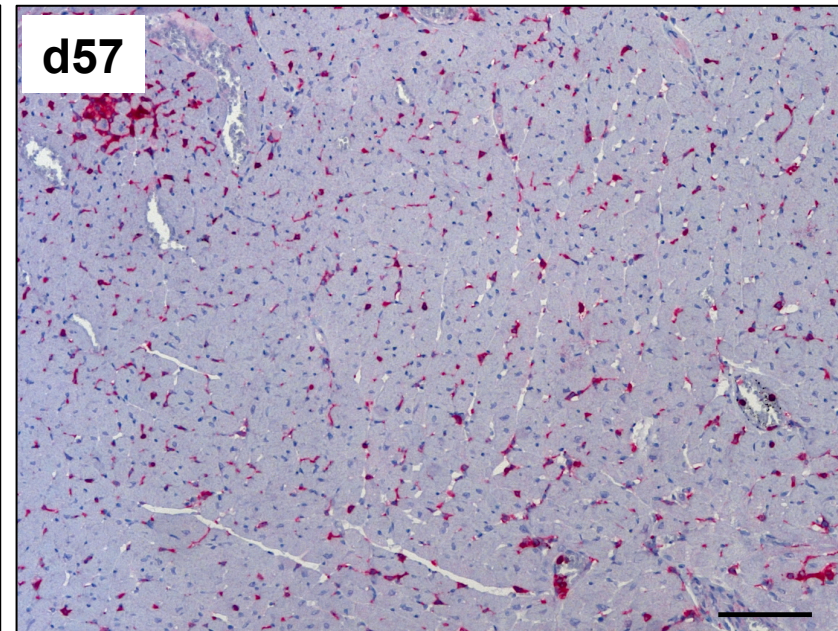

**C**

## **B Lymphocytes (B220+) - HEART**

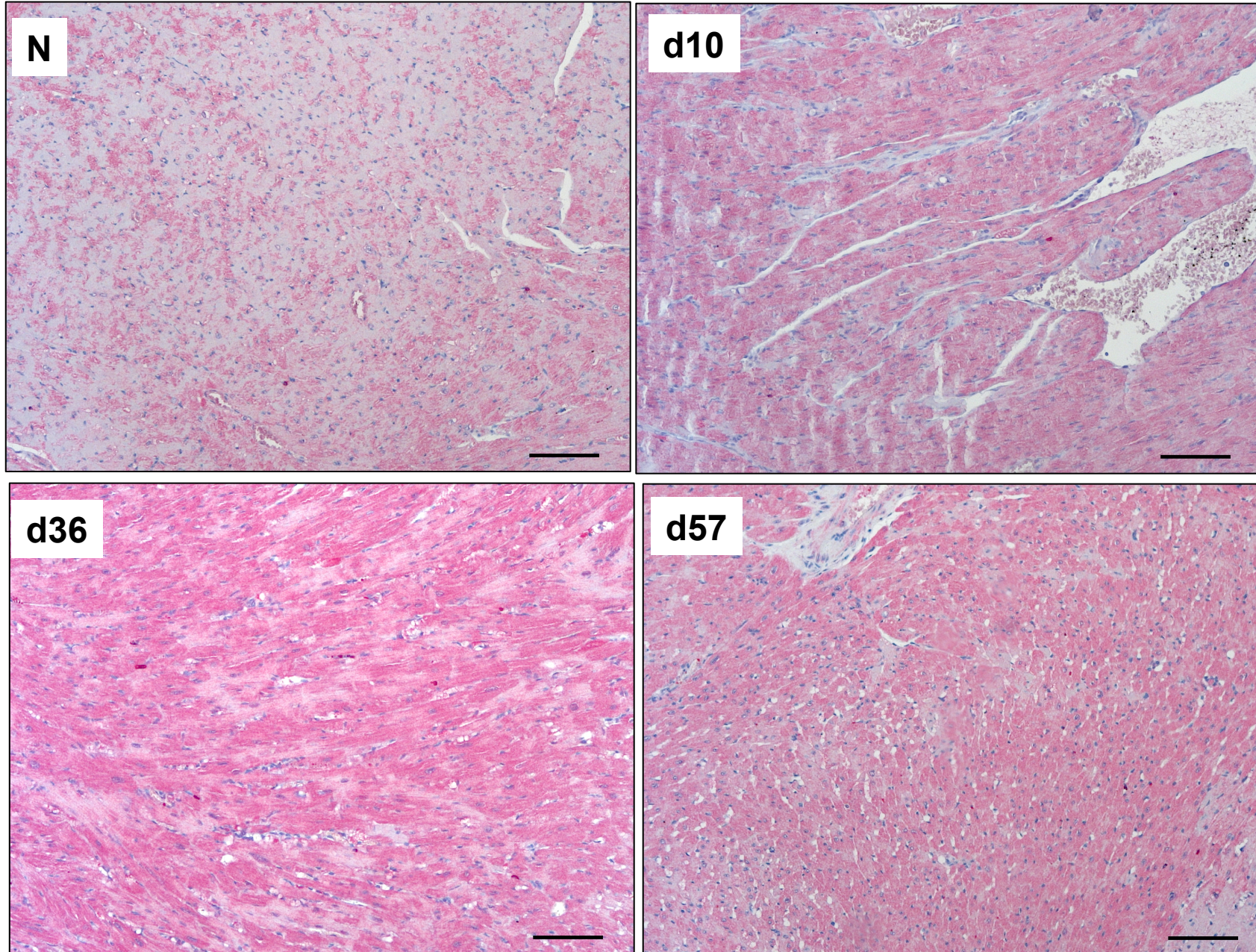

Supplement: Figure S6 — Microscopic inflammatory sequelae in the heart muscle over time following peroral low-dose T. gondii infection. Mice were perorally infected with one cyst of T. gondii on day 0 and surveyed at days (d) 10, 36, and 57 post-infection (p.i.; closed circles). Naive (N) mice served as uninfected controls (open circles). Out of six representative high power fields (HPF, 400x magnification) per animal the average numbers of (A) apoptotic (casapse3+, Casp3+) cells, of (B) T lymphocytes (CD3+) and (C) B lymphocytes were assessed in immunohistochemically stained cardiac paraffin sections. Medians (black bars), levels of significance (p-values) as determined by one-way ANOVA test followed by Tukey post-correction for multiple comparisons and numbers of analyzed mice (in parentheses) are indicated. Data were pooled from four independent experiments. [file Data_Sheet_6.PDF]

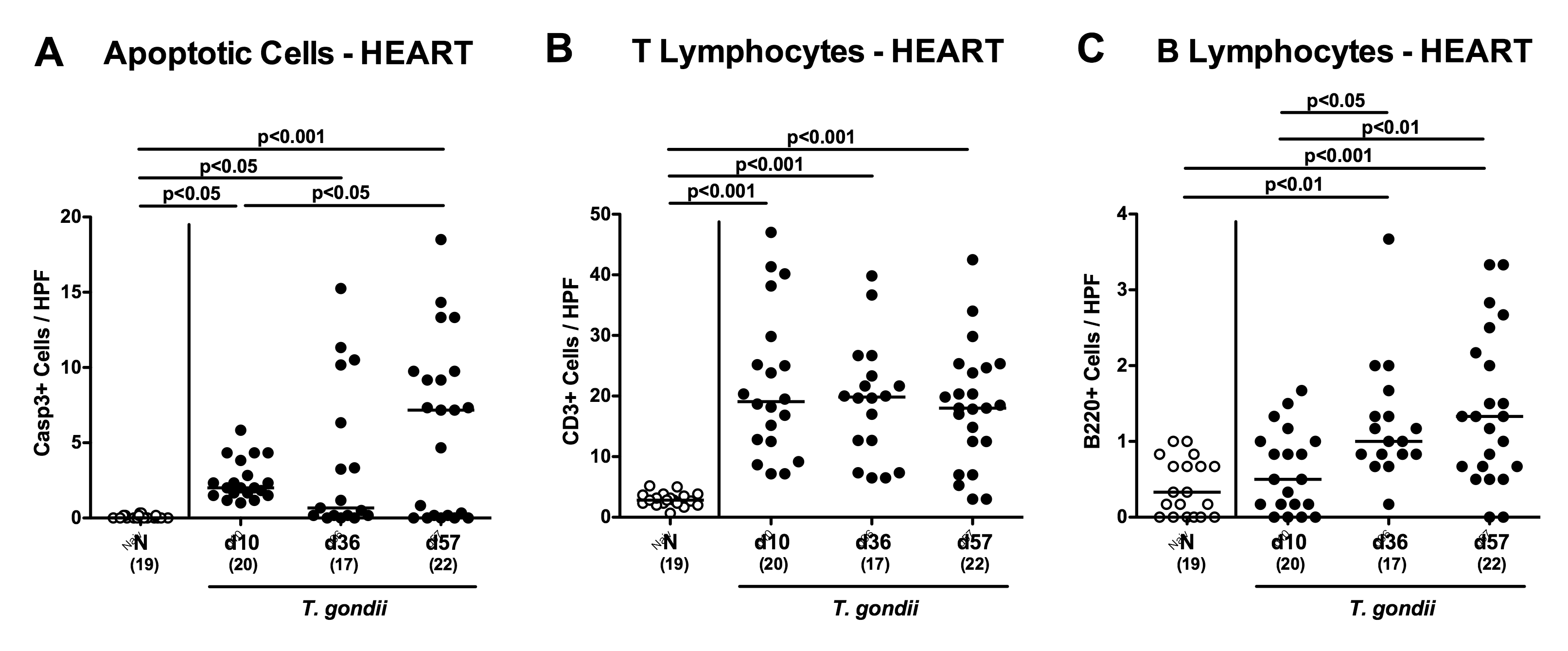

Supplement: Figure S7 — Representative photomicrographs illustrating inflammatory changes and distinct immune cell populations in the heart over time following peroral low-dose T. gondii infection. Mice were perorally infected with one cyst of T. gondii on day 0 and surveyed at days (d) 10, 36, and 57 post-infection (p.i.). Naive (N) mice served as uninfected controls. Representative photomicrographs taken from the heart illustrate (A) apoptotic (caspase3+, Casp3+) cells, (B) T lymphocytes (CD3+), and (C) B lymphocytes (B220+) in immunohistochemically stained paraffin sections at respective time points (100 x magnification, scale bar 100 μm). [file Image_1.TIFF]
